# On the Origin and Evolutionary History of *NANOG*

**DOI:** 10.1371/journal.pone.0085104

**Published:** 2014-01-17

**Authors:** Pierluigi Scerbo, Gabriel V. Markov, Céline Vivien, Laurent Kodjabachian, Barbara Demeneix, Laurent Coen, Fabrice Girardot

**Affiliations:** 1 Département Régulations, Développement et Diversité Moléculaire, Muséum National d'Histoire Naturelle, Centre National de la Recherche Scientifique, Paris, France; 2 Institut de Biologie du Développement de Marseille, Aix-Marseille Université, Centre National de la Recherche Scientifique, Marseille, France; 3 Institut de Génomique Fonctionnelle de Lyon, Université de Lyon, Ecole Normale Supérieure de Lyon, Centre National de la Recherche Scientifique, Lyon, France; 4 Department for Evolutionary Biology, Max-Planck-Institute for Developmental Biology, Tuebingen, Germany; 5 WatchFrog S.A., Evry, France; Wellcome Trust Centre for Stem Cell Research, United Kingdom

## Abstract

Though pluripotency is well characterized in mammals, many questions remain to be resolved regarding its evolutionary history. A necessary prerequisite for addressing this issue is to determine the phylogenetic distributions and orthology relationships of the transcription factor families sustaining or modulating this property. In mammals, the NANOG homeodomain transcription factor is one of the core players in the pluripotency network. However, its evolutionary history has not been thoroughly studied, hindering the interpretation of comparative studies. To date, the NANOG family was thought to be monogenic, with numerous pseudogenes described in mammals, including a tandem duplicate in Hominidae. By examining a wide-array of craniate genomes, we provide evidence that the NANOG family arose at the latest in the most recent common ancestor of osteichthyans and that *NANOG* genes are frequently found as tandem duplicates in sarcopterygians and as a single gene in actinopterygians. Their phylogenetic distribution is thus reminiscent of that recently shown for Class V POU paralogues, another key family of pluripotency-controlling factors. However, while a single ancestral duplication has been reported for the Class V POU family, we suggest that multiple independent duplication events took place during evolution of the NANOG family. These multiple duplications could have contributed to create a layer of complexity in the control of cell competence and pluripotency, which could explain the discrepancies relative to the functional evolution of this important gene family. Further, our analysis does not support the hypothesis that loss of *NANOG* and emergence of the preformation mode of primordial germ cell specification are causally linked. Our study therefore argues for the need of further functional comparisons between *NANOG* paralogues, notably regarding the novel duplicates identified in sauropsids and non-eutherian mammals.

## Introduction

In mammals, early embryonic cells are pluripotent as they can give rise to all embryonic cell lineages, but not to extra-embryonic tissues. This property is maintained in cell lines derived from embryos (Embryonic Stem Cells, ESCs) or reprogrammed by various strategies (induced Pluripotent Stem Cells, iPSCs) [1], [2]. Attempting to understand the extent to which the mammalian concept of pluripotency can be applied to other vertebrates is a classical problem of searching for homology, taking into account the fact that it could be uncoupled at different levels of biological organization [3]. A necessary step in this search for homology is the clarification of the evolutionary trajectories of the various molecular players implicated. However, establishing orthology relationships is not sufficient to infer functional conservation of orthologous proteins in distinct organisms. Even between relatively closely related species such as human and mouse, orthologous proteins can perform different functions [4]. Thus, structural conservation does not imply functional conservation, but to carry out functional comparisons on a safe basis, knowledge of the evolutionary history of gene families is necessary.

NANOG and Class V POU domain transcription factors are central to the network that controls pluripotency in mammals and are structurally conserved in osteichthyans. However, their functional conservation has been questioned.

The Class V POU domain family was initially thought to contain a single gene, called *pou2* in teleosts [5] and *POU5F1* (or *OCT4*) in eutherians [6], [7]. The discovery that monotremes and marsupials possess two paralogues, one more closely related to *pou2s* and the other to *POU5F1s*, revealed that gene duplication occurred in the evolutionary history of this family. The exact position of this duplication event in the vertebrate lineage was under debate [2], [8], [9], [10], [11], [12] until its recent clarification by Frankenberg and Renfree, who demonstrated that it predates the gnathostome radiation [13]. Resolving the controversial evolutionary relationship between teleost *pou2* and tetrapod *POU2* and *POU5F1* provided the framework for interpreting the functional data. *POU5F1s* and *POU2s* are believed to share common functions: controlling the timing of cell differentiation during development and being able to induce pluripotency in a mammalian iPSC assay [2]. Moreover, mammalian *POU5F1* can substitute for *pou2* during zebrafish development [12]; conversely, oct91, a *pou2* orthologue from *Xenopus laevis* or *POU5F1* from platypus are both able to efficiently replace *POU5F1* in mammalian ESCs [9], [14]. However, some functional diversification might have occurred, as *POU5F1* from axolotl, as well as *POU2* orthologues from opossum, chick and zebrafish are unable to fully replace *POU5F1* function in pluripotency maintenance in a mammalian ESC assay [2]. Similarly, *POU5F1* and *POU2* expression profiles suggest further functional differences concerning their role in development and germ-lineage specification [13].

*NANOG*, which was initially thought to be restricted to mammals, is considered to be present as a single orthologue in all osteichthyans, except *Xenopus* species, that seem to have lost this gene [15], [16]. Single *NANOG* genes have been described in eutherians, birds, axolotl and teleosts [14], [15], [17], [18], [19]. Remarkably, a duplicate, called *NANOGP1* or *NANOG2*, has been detected in hominids. It is thought to be an unprocessed pseudogene issued by tandem duplication [20], [21]. *NANOGP1* and other pseudogenes identified in primates and rodents are known to be expressed and functional [20], [22], [23]. Functional complementation data suggest that, among amniotes, *NANOG* biochemical properties are conserved. Indeed, overexpression of chick *Nanog* maintains pluripotency of mammalian LIF-deprived ESCs [14], [19], However, in its native form, the axolotl *NANOG* orthologue is unable to maintain pluripotency and self-renewal in LIF-deprived mouse ESCs, but gains these properties upon addition of a dimerization domain, derived from the mouse orthologue [15]. As to zebrafish *Nanog*, a first study reported that it is able to maintain pluripotency and self-renewal when ectopically expressed in LIF-deprived mouse ESCs — albeit with lower efficiency than mouse and chick orthologues — [19], while another concluded that it is not able to do so [24]. Other assays nevertheless argue in favor of the ability of zebrafish *Nanog* to regulate pluripotency in heterologous mammalian systems such as iPSCs induction [19] and embryoid body differentiation [18]. Reciprocally, human or mouse *Nanog* orthologues are able to rescue *Nanog* loss-of-function in zebrafish embryos [18], [24]. Contrasting with these observations, teleost *Nanog* genes do not appear to serve any pluripotency-related activity during endogenous embryogenesis, but could share functions in germ-line development with their mammalian counterparts [17], [24], [25].

We set out to improve our knowledge of the evolutionary history of the NANOG family, using approaches similar to those used for the Class V POU domain family by Frankenberg and Renfree [13]. We have identified novel *NANOG* paralogues and show that they are frequently found as tandem duplicates in sarcopterigyans. We propose that these duplicates are the product of at least four independent duplication events, rather than a single ancestral one as reported for Class V POU domain family.

## Methods

Known *NANOG* orthologues were retrieved from public repositories (Genbank and Ensembl) and BLAST (tblastn) searches were performed against available genomes and/or transcriptomes using the most conserved regions (encoded by the 2^nd^ and 3^rd^ exons, including the homeobox) of zebrafish, axolotl, chick, opossum and mouse NANOG proteins as queries. The screened dataset was chosen so as to ensure the broadest taxonomic range among craniates (including cyclostomes and chondrichthyans). In some cases (highlighted in red in Table S1) novel genes, pseudogenes or exons were identified. For those genes, putative translation start sites and exon-intron boundaries were assessed compiling automated predictions from GENSCAN [26], FGENESH [27] and/or NNSPLICE 0.9 [28] and then manually refined on the basis of the protein sequence alignment (see below). The sources for known or novel all gene models used in this study are listed in Table S1. Known or predicted protein sequences were aligned using the Muscle algorithm [29] with default parameters in Seaview [30]. The resulting alignment was manually curated, mainly in regions encoded by the least conserved 1^st^ and 4^th^ exons (File S1). Further, given that the 1st and 4^th^ exons of a significant number of *NANOG* paralogues were not retrieved (due to limitations in available data and/or low conservation), phylogenetic analyses were performed on a restricted protein alignment encompassing the region encoded by the 2^nd^ and 3^rd^ exons of the retrieved sequences (Figure S1).

**Figure 1:**
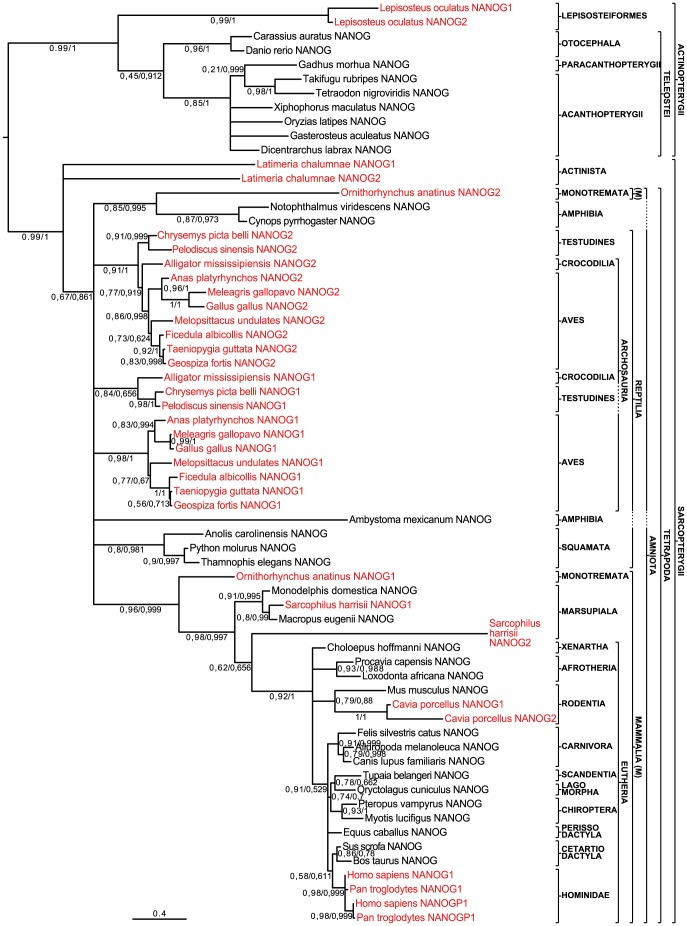
Phylogenetic analysis of NANOG paralogues in osteichthyans. Translated sequences of exons 2 and 3 were analyzed by Maximum Likelihood and Bayesian inference. Here we show a strict consensus tree, using the scaffold of the ML tree. Branch support was assessed and is given next to the relevant branches (top number: aLRT for the ML analysis and bottom number PP for the Bayesian analysis). The actinopterygian sequences form a monophyletic group that was used to root the tree. Duplicates are highlighted in red. Note that two paralogues are found in coelacanth (*Latimeria chalumnae*), Archosaurs (*Alligator mississipiensis*; *Gallus gallus*; *Meleagris gallopavo*; *Taeniopygia guttata*; *Melopsittacus undulates*; *Anas platyrhynchos*; *Geospiza fortis*; *Ficedula albicollis*), Testudines (*Pelodiscus sinensis*, *Chrysemys picta belli*), platypus (*Ornithorhynchus anatinus*), Tasmanian devil (*Sarcophilus harrisii*), Guinea pig (*Cavia porcellus*), Hominidae (*Pan troglodytes*; *Homo sapiens*) and spotted gar (*Lepisosteus oculatus*). The topology suggests that independent duplication events occurred in the three latter clades.

For Maximum Likelihood (ML) analysis, we used the PHYML version implemented in Seaview [30] with the JTT substitution model, 4 substitution rate categories, and estimated gamma distribution parameters. Tree searching relied on NNI with 10 random starting trees. Branch support was assessed using the aLRT SH-like method. For the Bayesian analysis, we used MrBayes version 3.2 [31], using the JTT substitution model. Four heated chains were run for 10 million generations, the cold chain being sampled at intervals of 10 000 generations. After discarding the first 2.5 million generations as burn-in, the remaining trees were used to generate a 50% consensus tree were branch support values were indicated as posterior probabilities (PP). Figure 1 gives a strict consensus of the ML and Bayesian trees, using the topology of the ML tree as a backbone, with aLRT and PP support values given for nodes that were recovered in both topologies.

**Figure 2:**
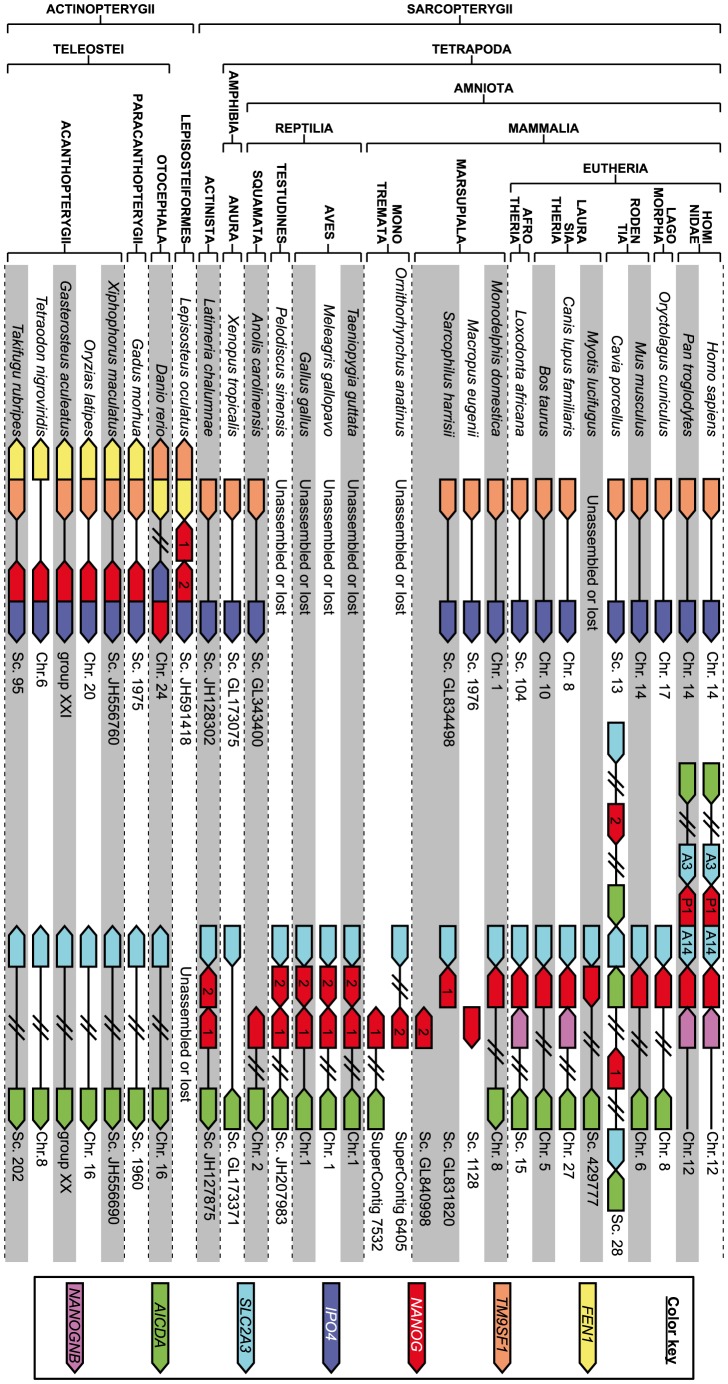
Simplified synteny of *NANOG* loci in osteichthyans. The synteny of the loci where *NANOG* orthologues are found in actinopterygians (*TMSF9* - *IPO4* region) and sarcopterygians (*SLC2A3* - *AICDA* region) are shown on the left-hand and right-hand sides, respectively. The relevant chromosomes or gene scaffolds are given. The figure is not drawn to scale, “empty” spaces along the chromosomes (e.g. between *TM9SF1* and *IPO4* in sarcopterygians) do not reflect actual distances but are meant to facilitate comparisons. Double slashes (//) denote that intervening genes were omitted for simplicity (e.g. between *FEN1* and *IPO4* in *Danio rerio*). In species in which two *NANOG* paralogues were found, numbers indicate which paralogue was named “*NANOG1*” or “*NANOG2*” in this work (note that these names do not imply orthologous relationships). This region contains multiple paralogues of *NANOG*, *AICDA* and *SLC2A3* in Guinea pig; and of both *NANOG* (*NANOGP1*, *P1* on the figure) and *SLC2A3* (named *SLC2A14*, *A14* on the figure) in Hominidae. More detailed information regarding these two regions is listed in File S2, including coordinates for the genes presented on this figure.

For synteny analysis, we first conducted an overview of the regions annotated in Ensembl release 70 using the genomicus server [32] and manually refined or retrieved the data for species for which the current state of genome annotation was inadequate for our purpose. In these species, orthology relationships of genes situated in the two conserved loci studied were assessed on the basis of trees provided by Treefam [33] or the EnsemblCompara Genetrees [34] when available, or by checking reciprocal best hits using blastp (with default parameters) between closely related species. The data thus generated is listed in File S2 and summarized in Figure 2.

Positive selection was tested using the branch-site model A, as implemented in codeml from the PAML package version 4b [35]. Positive selection is detected if there is a category of sites with dN/dS ratio omega >1 on the tested branch. Importantly, the test contrasts positive selection on the branch of interest to the possibility of relaxed purifying selection, which avoids a major source of false positive results. The test is done by comparing the difference of log-likelihood (lnL) values to a chi2 distribution of 1 degree of freedom and corrected for multiple testing [36]. The test was carried on the whole homeobox (180 nucleotides) on a representative set of vertebrates. Duplicates for which there are no functional data, including expression data, were removed in order to restrict the analysis to sequences for which the nucleotidic sequence is more reliable. An exception was made for the coelacanth, because it is not formally possible to distinguish between the two duplicates, and the sequences are informative due to their phylogenetic position.

## Results

A Blast-based approach followed by de novo gene prediction led to the identification of novel *NANOG*-like genes in the spotted gar (2), coelacanth (2), Indian python (1), American alligator (2), medium ground finch (2) and platypus genomes (1 novel and 1 already predicted). Further, gene models not annotated as belonging to the NANOG family were retrieved from the painted turtle (2), budgerigar (2), duck (2), collared flycatcher (2), Tasmanian devil (1 novel and 1 already annotated) and Guinea pig (1 novel and 1 already annotated) genomes (see Table S1). The deduced amino acid sequences thus identified were compiled and aligned with known NANOG sequences obtained from public repositories, some of which were improved by de novo predictions of intron-exon boundaries and/or transcription start sites, as well as by manual curation (File S1). Overall, two putative *NANOG* genes were detected in most sarcopterygians, including the coelacanth, Archosauria, Testudines, Hominidae, Guinea pig, platypus and Tasmanian devil as well as in the spotted gar. In contrast, no clear *NANOG*-related sequence was retrieved from chondrichthyans, cyclostomes, urochordates or cephalochordates. To understand the phylogenetic relationships between these genes, we performed Maximum Likelihood and Bayesian reconstructions based on the protein sequences translated from the 2^nd^ and 3^rd^ exons, which include the whole homeobox (Figure S1). On the basis of previous studies [16], we rooted the resulting strict consensus tree on the branch separating the actinopterygians from the sarcopterygians (Figure 1). The observed topology shows conservation in teleosts and a complex pattern of gene duplications in other gnathostomes. All actinopterygian and sarcopterygian sequences form two monophyletic groups. In the sarcopterygian group, mammals, theria, eutheria, afrotheria, carnivora and hominidae sequences are monophyletic. Among actinopterygian sequences, euteleostei, neoteleostei, cyprinidae and tetraodontiformes form monophyletic groups.

Three groups interpreted relatively straightforwardly. First, both spotted gar paralogues form a strongly supported monophyletic group at the base of the monophyletic actinopterygian NANOG sequences. This topology suggests that a duplication event occurred in the spotted gar lineage after its divergence from the rest of the actinopterygians. Second, human and chimp NANOGP1 paralogues form a monophyletic group arising among their respective cognate paralogues. This topology is coherent with previous work showing that these genes arose through a duplication that occurred in a Hominidae ancestor [21]. Last, the two Guinea pig paralogues form a strongly supported monophyletic group, suggesting that another independent duplication event took place in this lineage.

Other groups are more problematic. The sequences from urodeles do not form a monophyletic clade. The axolotl sequence is found at an unresolved basal position among amniote species, whereas the newt sequences form a monophyletic group with one platypus paralogue, at the unresolved base of sarcopterygians. In Reptilia, two paralogues were detected in all archosaurs and testudines studied, while only one was retrieved from squamate genomes. These sequences fall in four monophyletic groups. The first group gathers with strong support (0.91 aLRT, 1.00 PP) one paralogue from each studied archosaur and testudines species (NANOG2). The second group (0.98 aLRT, 1 PP) contains the second paralogue from birds (NANOG1) and the third group (0.94 aLRT, 1 PP) contains the second paralogue from turtles and alligator (NANOG1). Squamate NANOG sequences compose a fourth monophyletic group (0.8 aLRT, 0.981 PP). The relationships between these four groups are unresolved among sarcopterigians, but all NANOG1 sequences form a monophyletic group in the ML tree (aLRT 0,78, collapsed in the Bayesian reconstruction). A possible interpretation is that a single *NANOG* gene was present in the most recent common ancestor of the Reptilia and that this ancestral condition is conserved in Squamata, while a duplication event took place in a common ancestor of Testudines and archosaurs [37], leading to the two paralogs found in these clades. Two sequences were retrieved from the sole monotreme species studied, the platypus, one of which (NANOG2) groups together with urodele sequences (*Notophtalmus viridescens* and *Cynops pyrrhogaster*) at the base of the sarcopterygian genes. This unexpected basal position could be attributed to long-branch attraction, a bias of phylogenetic reconstruction whereby highly divergent sequences tend to be grouped together in an artefactual basal position [38]. Out of 67 conserved positions, platypus NANOG2 shares 36 and 37 identical sites with *Notophtalmus viridescens* and with *Cynops pyrrhogaster* NANOG sequences, respectively (53.75% and 55.22% identity), it is therefore no more similar to these amphibian sequences than to other non-mammalian sequences, since it shares 37 sites with coelacanth NANOG1 and NANOG2 from painted turtle, American alligator, medium ground finch, zebra finch and collared flycatcher (55.22% identity) and 38 sites with budgerigar NANOG2 (56.71% identity), which is the most similar sequence among those studied. However, platypus NANOG2 is clearly less similar to mammalian NANOG sequences, the most similar being Guinea pig NANOG1 (35 sites, 52.23% identity) and, importantly, it shares only 34 positions with platypus NANOG1 (50.74% identity). The high divergence of platypus NANOG2 could be due to pseudogenization, which might follow gene duplication [39]. The second platypus paralogue (NANOG1) is found at the base of the mammalian NANOGs, forming the sister-group of the marsupial and eutherian group. Among the three marsupial species studied, only the Tasmanian devil displayed two putative NANOG genes. One (NANOG1) groups with other marsupial sequences (0.91 aLRT, 0.995 PP), whereas the second (NANOG2) forms a weakly supported group with eutherian sequences (0.62 aLRT, 1 PP). The monophyly of eutherian NANOGs is strongly supported (0.92 aLRT, 1 PP). Last, the coelacanth duplicates occupy an unresolved basal position among sarcopterygians. Therefore, our data supports the idea that *Nanog* has a monogenic origin (i.e. it was present as a single gene in the most recent common ancestor of the osteichthyans) [19] and was subsequently duplicated independently in diverse lineages during the evolution of bony vertebrates. However, except for the three cases underlined above, namely the spotted gar, hominids and Guinea pig, phylogenetic reconstruction alone is not sufficient to resolve unambiguously the evolutionary history of duplications among NANOG paralogs.

In order to clarify orthology relationships between *NANOG* duplicates, we next analyzed their synteny in available osteichthyan genome assemblies. In actinopterygians, all *nanog* paralogs are found closely associated with *TM9SF1* and *IPO4*. Conversely, in all sarcopterygians, including the coelacanth, *NANOG* paralogs are found in a region containing *SLC2A3* and *AICDA* (Figure 2 and File S2). *Xenopus tropicalis* constitute the sole clear exception, since *NANOG* has been lost in this species (and probably in *X. laevis* as well; [16], [18]). *Sus scrofa* might be another possible exception to this syntenic conservation. Indeed, in the available pig genome sequence (ensembl release 72), the *NANOG* gene is annotated as being located on chromosome 1, whereas the conserved syntenic region is located on chromosome 5. However, this gene is clearly a product of retrotransposition, since it does not possess any intron. Remarkably, numerous assembly gaps are present in this genomic region in pig, including a 50,000 bp-long one immediately downstream of the *SLC2A3* orthologue. Further, mapping of a clone encompassing the second exon of *NANOG* to pig chromosome 5 has been reported [49].

Thus, as previously reported, the chromosomal synteny of *NANOG* is not conserved between actinopterygians and sarcopterygians [17], [18], [19]. This observation suggests that a translocation event occurred early in the evolutionary history of this gene, probably in the actinopterygian stem lineage, before the 3R whole-genome duplication specific to this clade [19]. Both spotted gar paralogs are found as direct tandem duplicates at the position where all actinopterygian *NANOG* genes are found, which is consistent with the hypothesis that they arose from a duplication event specific to this lineage. Similarly, the fact that the duplication already described in the Hominidae clade encompasses the neighboring *SLC2A3* loci (giving rise to *SLC2A14*, also restricted to hominids, see Figure 2) reinforces the notion that this event is specific to this lineage. The Guinea pig locus displays a more complex organization, which suggests that at least two segmental duplications took place in the region leading to multiple local duplicates, not only of *NANOG*, but also –among others– of *SLC2A3* and *AICDA* (Figure 2 and File S2). This organization clearly supports the notion that an independent duplication event also took place in a Guinea pig ancestor. In archosaurs and turtles, *NANOG* paralogs are found in the same *SLC2A3* - *AICDA* region, as inverted tandem duplicates. This common genome organization strongly supports the concept that paralogs from archosaurs and turtles form two orthologous groups and result from another distinct duplication event. The single *NANOG* paralog retrieved from opossum and one of the paralogs from Tasmanian devil are found at the same *SLC2A3* - *AICDA* genomic location (a putative pseudogene is located distantly upstream on the same chromosome in the opossum, see File S2), whereas the second Tasmanian devil paralog, as well as the only wallaby ortholog identified, are both the sole genes present on their short genomic scaffolds, precluding synteny analysis (Figure 2 and File S2). Note that the single wallaby ortholog identified in our study corresponds to the sequence used to raise the NANOG antibody reported in [40]. In platypus, both paralogs are found at the edge of separate scaffolds, one also bearing a *SLC2A3* ortholog, the other an *AICDA* ortholog. It is therefore possible that both platypus genes form a direct tandem repeat, ruptured by incomplete assembly. Last, both coelacanth orthologs form a direct repeat in the same genomic region. Therefore all sarcopterygian duplicates, for which synteny could be unambiguously assessed, are located in a conserved genomic position (Figure 2 and File S2). Further, in some eutherians, another homeodomain-encoding gene, *NANOGNB*, forms a direct tandem with *NANOG*, reminiscent of the organization observed in other species possessing two *NANOG* paralogs (Figure 2).

The evolutionary history of *NANOG* is therefore difficult to reconstruct based on the available data. We propose a putative scenario in which *NANOG* arose as a single gene in an osteichthyan ancestor. This ancestral monogenic state would have been retained in actinopterigyans (except in the spotted gar, see below). We cannot determine if a duplication took place in the sarcopterygian stem lineage, with both duplicates being retained in the coelacanth, and one lost in tetrapods, or if a tandem duplication occurred specifically in the coelacanth lineage. Nonetheless, the last common ancestor of extant tetrapods would have possessed a single *Nanog* gene. This gene would have been duplicated in a common ancestor of Archosaurs and Testudines. In mammals, a duplication event might have occurred in the stem lineage, one paralogue having highly diverged in platypus, but being retained in Marsupials and lost in Eutheria. Alternatively, both *NANOG2* paralogues from Tasmanian devil and platypus might have arisen from independent duplications. Last, independent segmental duplications encompassing *SLC2A3* and *NANOG* would have taken place in the hominid and Guinea pig lineages, whereas a duplication event restricted to NANOG would have occurred in the spotted gar lineage.

## Discussion

We retrieved two *NANOG* paralogues from most sarcopterygian and one actinopterygian genomes studied. Phylogenetic reconstruction and synteny analysis suggest that multiple tandem duplications have taken place in the evolutionary history of the *NANOG* family. Four such events are unambiguously supported by the topology of the tree presented here: one in the spotted gar lineage, one before the diversification of the Archosauria/Testudines clade, one in the hominid lineage and one in a Guinea pig ancestor. The unresolved status of the novel putative paralogues in monotremes and marsupials precludes any definitive conclusion concerning their orthology relationships and may hinder the interpretation of the pattern of duplications and losses in amniotes. The same restrictions apply to coelacanth paralogues. In amphibians, the *NANOG* family also displays surprising features. Indeed, the gene has been lost in *Xenopus*, making them unique in this respect amongst osteichthyans [16], [18]. Further, the sole *NANOG* gene known in axolotl does not group with other urodele homologues, represented by newts. It should be kept in mind that no complete urodele genome is available to date, and that urodele genomes are the largest in size among tetrapods [41]. Thus, it is possible that known axolotl and newts *NANOG* genes are only one of multiple paralogues. This uncertainty should be resolved by a more comprehensive exploration of their genomes. An additional factor that could lead to even greater structural diversity in the *NANOG* family is gene conversion between tandem paralogues [42], [43]. If this were the case, the signature of an ancient duplication would have been lost by a recombination event between two adjacent paralogues, which would artefactually appear as convergent recent duplicates. However, this would not change our main conclusion that the complex evolutionary history of *NANOG* argues for additional functional studies before inferring causal links between distinct evolutionary patterns and phenotypes.

Three lines of evidence support the idea that a functional shift took place during *NANOG* evolutionary history. First, zebrafish *Nanog* has been reported to be unable to sustain LIF-independent self-renewal when overexpressed in mouse ES cells [24], or to do so with less efficiency than amniote orthologues [19]. Second, we have shown that orthologues from teleost (*OlNanog*) and amniote (*mNanog*) have distinct effects when overexpressed in developing *Xenopus laevis* embryos, although both seem to perturb tissue morphogenesis (see supplemental data in [16]). Third, testing for positive selection in the *NANOG* group revealed a significant signal for relaxation of selection in the branch separating actinopterigyans and sarcopterygians (Figure S2). Given the absence of a reliable outgroup, it is not possible to determine whether this evolutionary event occurred in the sarcopterygian or actinopterygian stem lineage. Nevertheless, zebrafish *Nanog* can unambiguously replace mouse *Nanog* in the reprogramming process [19] and protect murine embryoid bodies from differentiation [18]. Reciprocally, mouse and human *NANOG* can replace zebrafish *Nanog* during early teleost embryogenesis [18], [24]. Therefore, NANOG proteins have retained an ancestral transcriptional activity in these species and this conserved function is sufficient to achieve reprogramming, whereas efficient *in vitro* maintenance of ESCs might be an exclusive property of amniote NANOG orthologues [14], [24]. Intriguingly, the native axolotl NANOG orthologue also seems to lack bona fide (or amniote-like) pluripotency maintenance activity, but the addition of a dimerization domain to the protein allows it to acquire this activity [15].

Altogether, these considerations raise the question of the nature of the ancestral function(s) of *NANOG* in the osteichthyan, actinopterygian, tetrapod and amniote last common ancestors. In chick, tammar wallaby and placental mammals, *NANOG1* and *NANOG*, respectively, are expressed in uncommitted epiblastic cells, whereas their expression becomes undetectable in cells undergoing differentiation and epithelial-mesenchymal transition during gastrulation [14], [40]. None of the *NANOG2* paralogues from sauropsids described in this work have been functionally studied to date, leaving open the issue of a possible diversification between *NANOG* paralogues in this clade. The same argument can be applied to the platypus and Tasmanian devil paralogues. In addition, given the important role played by NANOG dimers in mammals (reviewed in [44]), the possibility of heterodimerization of NANOG proteins in species that possess two paralogues should be explored. Another possibility is that in species where two *NANOG* paralogues coexist these additional genes have a quantitative impact on pluripotency networks. In parallel, teleost *NANOG* orthologues do not seem to regulate pluripotency in vivo but to control some aspects of cell migration and embryonic morphogenesis [24], [25], [45]. Remarkably, NANOG is also implied in the control of cell migration and behavior in mammalian systems [45], [46]. Thus, the control of cell migration and behavior may be the most ancient and widely conserved function of *NANOG*, whereas it would have acquired a novel function specific to sarcopterygians (or tetrapods, or amniotes) in pluripotency. It is tempting to correlate such a functional innovation to the structural variations created by the gene translocation already reported [19] and/or to the tandem duplications revealed in this study. Functional studies of other *NANOG* paralogues are clearly needed to shed light on these issues. In particular, assessing the properties of the Coelacanth, spotted gar and other urodeles paralogues in heterologous systems such as *Xenopus* embryos, chick embryos and ES cells as well as mammalian ES and iPS cells would be highly informative.

Another point is that our data unequivocally show that some sauropsids (i.e. birds and alligator), which are believed to specify primordial germ cells (PGCs) via inherited germ plasm retain not only one, but two *NANOG* paralogues in their genome. Therefore acquisition of the preformation mode of germ cell specification does not imply loss of *NANOG*. While not contradicting it, this observation does not support the hypothesis that loss of *NANOG* in *Xenopus* is linked to the emergence of maternally inherited germ plasm in anurans [15]. Similarly, before the *POU5F1*/*POU2* duplication event was elucidated it had been argued that the evolution of this gene family allowed the emergence of innovations relative to extraembryonic annexes and modes of PGC induction [9], [10], [47]. These hypotheses had to be reevaluated in the light of more comprehensive understanding of the evolutionary histories of Class V POU genes [10], [13]. These cases could serve as a warning that given the complexity of *NANOG* duplication patterns across vertebrates, and given the scarcity of functional data, it might be premature to draw conclusions about the causative role of those duplication events in relation to embryogenesis or germ line specification mode.

## Conclusions

In summary, both Class V *POU* and *NANOG* genes were present in the genome of the common ancestor of extant osteichthyans and are often found as small two-member families in a given species. They nevertheless display contrasted evolutionary histories. Class V *POU* genes have been subjected to an ancestral duplication, consistent with the pattern of whole genome duplications known to have occurred before the gnathostome radiation, followed by numerous specific losses of one or the other paralogue in various lineages [13]. In contrast, *NANOG* has been subjected to numerous independent duplications during the evolutionary history of osteichthyans. We hypothesize that the duplication and maintenance of *NANOG* genes could have contributed to create a layer of complexity in the control of cell competence and pluripotency. Noteworthy, our analysis does not support the causal link between emergence of a preformation mechanism to specify PGCs and loss of *NANOG*, as previously suggested [48]. In this light, more extensive functional analyses *in vivo* will be necessary to understand how *NANOG*, and Class *V POU* genes, might have contributed to greater plasticity and evolvability of developmental mechanisms, and thus to the diversity of embryonic developmental modes in vertebrates.

## Supporting Information

Figure S1 **Alignment used for the phylogenetic analysis.** The regions encoded by the 2^nd^ and 3^rd^ exons of the *NANOG* genes, as well the homeodomain are indicated below the alignment.
(PDF)

Figure S2 **Test for positive selection and branch relaxation during** ***NANOG*** **evolution.** A) Tree showing the three branches that were checked for positive selection. B) P-values for the likelihood-ratio test concerning the three tested branches. The only significant event detected is a relaxation of positive selection in the branch separating sarcopterygians and teleosts is significant.
(PDF)

File S1 **Full-length alignment of predicted protein sequences of known or novel** ***NANOG*** **paralogues in fasta format.** Fasta format files containing all the NANOG-related sequences retrieved in this study. The exon/intron boundaries are indicated by “X” residues.
(TXT)

File S2 **Listing of the loci surveyed for synteny analysis.** For each studied species, the relevant chromosome(s) or genomic scaffold(s) are indicated in bold, with the coordinates and orientation of the relevant genes listed below in italics. Double slash (//) indicates the presence of intervening genes that have been omitted for the sake of simplicity. Relevant genes that were not found to be located on the same chromosome/scaffold as *NANOG* orthologues are bracketed. Putative novel orthologues of relevant genes are indicated.
(PDF)

Table S1 **Table listing the source of the** ***NANOG*** **sequences used in this study.** Coordinates of the genes on chromosomes/genomic scaffolds are given when available. Genes that were either newly predicted or for which novel or revised intron/exons boundaries were identified are listed in red.
(DOCX)
