# Supplementary material for: On the Origin and Evolutionary History of NANOG
Source: PLoS One. 2014 Jan 17;9(1):e85104. doi: 10.1371/journal.pone.0085104 (PMC3894937; doi:10.1371/journal.pone.0085104)
Supplement: Figure S2 — Test for positive selection and branch relaxation during NANOG evolution. A) Tree showing the three branches that were checked for positive selection. B) P-values for the likelihood-ratio test concerning the three tested branches. The only significant event detected is a relaxation of positive selection in the branch separating sarcopterygians and teleosts is significant. (PDF) [file pone.0085104.s002.pdf]

A

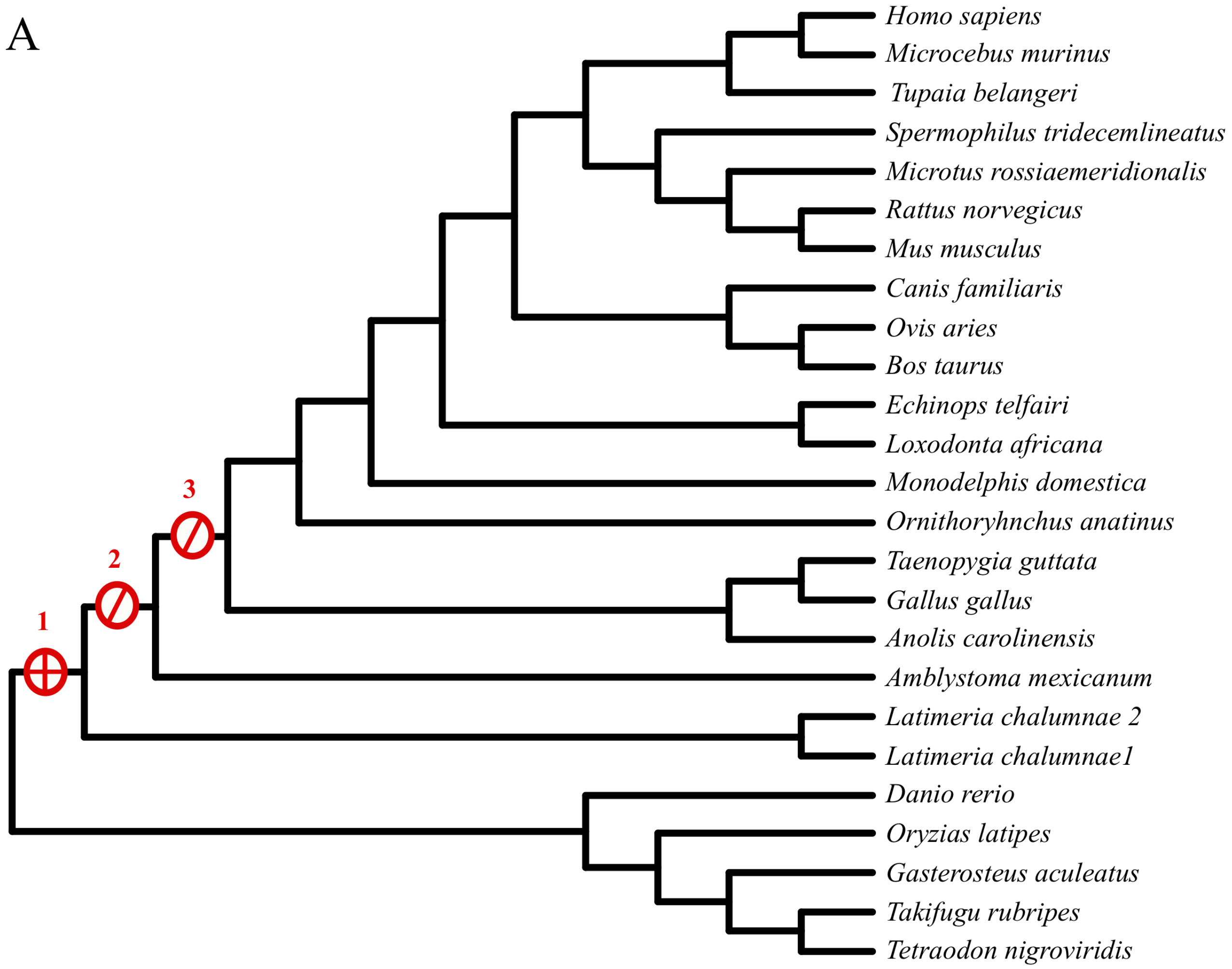

B

			Models			Likelihood Ratio Test	
			No shift	Branch relaxation	Positive selection	Relaxation	Positive selection
Tested branch	Species	Sites	lnL	lnL	lnL	P-value	P-value
3 - Tetrapodes	25	180	-2403.79	-2403.28	-2400.73	0.048 (NS)	0.024 (NS)
2- Amniotes	25	180	-2403.79	-2402.97	-2401.55	0.106 (NS)	0.092 (NS)
1- Teleosts vs Sarcopterygians	25	180	-2403.79	-2392.84	-2389.69	7.524e-07***	0.012
