# Supplementary material for: On the Origin and Evolutionary History of NANOG
Source: PLoS One. 2014 Jan 17;9(1):e85104. doi: 10.1371/journal.pone.0085104 (PMC3894937; doi:10.1371/journal.pone.0085104)
Supplement: File S2 — Listing of the loci surveyed for synteny analysis. For each studied species, the relevant chromosome(s) or genomic scaffold(s) are indicated in bold, with the coordinates and orientation of the relevant genes listed below in italics. Double slash (//) indicates the presence of intervening genes that have been omitted for the sake of simplicity. Relevant genes that were not found to be located on the same chromosome/scaffold as NANOG orthologues are bracketed. Putative novel orthologues of relevant genes are indicated. (PDF) [file pone.0085104.s004.pdf]

Actinopterigyan *NANOG* locus (based on Ensembl release 70)

Homo sapiens (Human) Chromosome 14
[PDCD6 Chromosome 5: 271,736-353,971 forward strand.]
--
REC8 Chromosome 14: 24,641,062-24,649,463 forward strand.
IPO4 Chromosome 14: 24,649,425-24,658,170 reverse strand.
TM9SF1 Chromosome 14: 24,658,349-24,682,679 reverse strand.
--
[FEN1] Chromosome 11: 61,560,109-61,564,716 forward strand.

Tupaia belangeri (Tree Shrew) GeneScaffold_5211
REC8 GeneScaffold_5211: 165,452-169,459 forward strand.
IPO4 GeneScaffold_5211: 169,779-178,054 reverse strand.
ENSTBEG00000010249 GeneScaffold_5211: 178,672-185,140 reverse strand.
*(**TM9SF1 ortholog**)*
--
[FEN1 GeneScaffold_1981: 62,833-63,975 forward strand.]

Canis lupus familiaris (Dog) Chromosome 8
[PDCD6 Chromosome 34: 11,910,120-11,929,648 reverse strand.]
--
REC8 Chromosome 8: 4,165,879-4,171,414 forward strand.
IPO4 Chromosome 8: 4,167,638-4,181,151 reverse strand.
TM9SF1 Chromosome 8: 4,180,758-4,201,106 reverse strand.
--
[FEN1 Chromosome 18: 54,681,760-54,682,902 reverse strand.]

Mustela putorius furo (Ferret) Scaffold GL896946.1
[PDCD6 Scaffold GL897032.1: 6,130,636-6,149,080 reverse strand.]
--
*TM9SF1 Scaffold GL896946.1: 6,577,316-6,586,153 forward strand. (**Unpredicted, putative**)*
IPO4 Scaffold GL896946.1: 6,597,016-6,605,853 forward strand.
REC8 Scaffold GL896946.1: 6,606,185-6,611,558 reverse strand.
--
[FEN1 Scaffold GL897050.1: 3,690,720-3,691,862 reverse strand.]

Loxodonta africana (Elephant) SuperContig scaffold_104
[PDCD6 SuperContig scaffold_169: 553,025-569,005 reverse strand.]
--
REC8 SuperContig scaffold_104: 2,838,690-2,844,632 forward strand.
IPO4 SuperContig scaffold_104: 2,844,979-2,855,865 reverse strand.
*ENSLAFG00000015869 SuperContig scaffold_104: 2,856,255-2,861,124 reverse strand. (**TM9SF1 ortholog**)*

Bos taurus (Cow) Chromosome 10
[PDCD6 Chromosome 20: 71,916,825-71,932,650 reverse strand.]

--
*BT.49827 Chromosome 10: 20,796,193-20,801,173 forward strand. (**TM9SF1 ortholog**)*
IPO4 Chromosome 10: 20,801,455-20,810,605 forward strand.
REC8 Chromosome 10: 20,810,613-20,816,425 reverse strand.
--
[FEN1 Chromosome 29: 40,931,652-40,936,454 forward strand.

Sus Scrofa (Pig) Chromosome 7
*[PDCD6 Scaffold GL896405.1: 25,993-42,468 reverse strand. (**Unpredicted, putative**)]*
--
*CHMP4A Chromosome 7: 80,356,854-80,381,322 forward strand. (**TM9SF1 ortholog**)*
IPO4 Chromosome 7: 80,381,676-80,389,841 forward strand.
REC8 Chromosome 7: 80,385,995-80,396,581 reverse strand.
--
[FEN1 Chromosome 2: 9,244,353-9,245,495 forward strand.]

Pterobat vampirus (Megabat) GeneScaffold_616
*[PDCD6 scaffold_9846:68-13130 reverse strand (**partial sequence**)]*
--
REC8 GeneScaffold_616: 1,662-8,059 forward strand.
IPO4 GeneScaffold_616: 8,409-16,583 reverse strand.
TM9SF1 GeneScaffold_616: 17,197-21,562 reverse strand.
--
[FEN1 GeneScaffold_70: 229,107-230,252 forward strand.]

Dipodomys ordii (Kangaroo rat) GeneScaffold_3331
REC8 GeneScaffold_3331: 51,063-59,800 forward strand.
IPO4 GeneScaffold_3331: 60,141-68,900 reverse strand.
TM9SF1 GeneScaffold_3331: 69,458-76,412 reverse strand.
--
[FEN1 GeneScaffold_2920: 66,963-68,105 forward strand.]

Mus musculus (Mouse) hromosome 14
[PDCD6 Chromosome 13: 74,303,121-74,317,326 reverse strand.]
--
REC8 Chromosome 14: 55,618,037-55,625,395 forward strand.
IPO4 Chromosome 14: 55,625,400-55,637,895 reverse strand.
TM9SF1 Chromosome 14: 55,635,965-55,643,806 reverse strand.
--
[FEN1 Chromosome 19: 10,199,132-10,204,169 reverse strand.]

Cavia porcellus (Guinea pig) scaffold_13
[PDCD6 scaffold_150: 1,933,703-1,953,559 reverse strand.]
--
TM9SF1 scaffold_13: 16,653,591-16,658,523 forward strand.

IPO4 scaffold_13: 16,659,142-16,667,422 forward strand.
REC8 scaffold_13: 16,667,745-16,674,000 reverse strand.
--
[FEN1 scaffold_65: 9,582,641-9,583,783 reverse strand.]

Oryctolagus cuniculus (Rabbit) Chromosome 17
[PDCD6 Scaffold GL018897: 325,073-343,786 reverse strand.]
--
REC8 Chromosome 17: 44,220,261-44,223,572 forward strand.
IPO4 CChromosome 17: 44,223,873-44,231,721 reverse strand.
*ENSOCUG00000002895 Chromosome 17: 44,232,011-44,239,019 reverse strand. (**TM9SF1 ortholog**)*
--
[FEN1 Scaffold GL018717: 3,013,810-3,014,952 reverse strand.]

Ictidomys tridecemlineatus (Squirrel) JH393532.1
[PDCD6 Scaffold JH393625.1: 1,129,814-1,143,945 reverse strand.]
--
TM9SF1 Scaffold JH393532.1: 341,421-360,926 forward strand.
IPO4 Scaffold JH393532.1: 360,250-369,455 forward strand.
REC8 Scaffold JH393532.1: 369,426-375,243 reverse strand.
--
[FEN1 Scaffold JH393391.1: 1,329,408-1,330,544 reverse strand.]

Monodelphis domestica (Opossum) Chromosome 1
[PDCD6 Chromosome 3: 88,822,085-88,852,701 forward strand.]
--
REC8 Chromosome 1: 173,515,823-173,528,843 forward strand.
IPO4 Chromosome 1: 173,522,649-173,537,522 reverse strand.
TM9SF1 Chromosome 1: 173,537,847-173,542,408 reverse strand.
--
[FEN1 Chromosome Un: 53,620,954-53,622,096 reverse strand.]

Macropus eugenii (Wallaby) GeneScaffold_1673
REC8 GeneScaffold_1673: 22,329-27,567 forward strand.
IPO4 GeneScaffold_1673: 27,917-43,833 reverse strand.
TM9SF1 GeneScaffold_1673: 44,381-48,637 reverse strand.
--
[FEN1 GeneScaffold_3672: 41,072-42,214 forward strand.]

Sarcophilus harrisii (Tasmanian devil) Scaffold GL834498
*[PDCD6 GL841201.1:3548294-3575715 forward strand. (**Unpredicted, putative**)]*
--
REC8 Scaffold GL834498.1: 32,176-36,864 forward strand.
IPO4 Scaffold GL834498.1: 37,187-54,118 reverse strand.
TM9SF1 Scaffold GL834498.1: 54,855-70,226 reverse strand.
--
[FEN1 Scaffold GL865337.1: 72,117-73,259 reverse strand.]

Ornithorhynchus anatinus (Platypus) (Lost, unsequenced or unnassembled region)
[PDCD6 UltraContig Ultra474: 820,999-842,057 reverse strand.]
--
[FEN1 UltraContig Ultra95: 474,865-477,717 reverse strand.]

Taeniopygia guttata (Zebrafinch) (Lost, unsequenced or unnassembled region)
[PDCD6 Chromosome 2: 94,131,409-94,140,946 forward strand._]
--
[FEN1 Chromosome 5: 6,910,279-6,911,394 forward strand.]

Meleagris gallopavo (Turkey) (Lost, unsequenced or unnassembled region)
[PDCD6 Chromosome 3: 38,246,457-38,251,723 forward strand.]
--
[FEN1 Chromosome 5: 6,368,029-6,369,174 reverse strand.]

Gallus gallus (Chick) (Lost, unsequenced or unnassembled region)
[PDCD6 Chromosome 2: 88,929,444-88,935,921 forward strand.]
--
[FEN1 Chromosome 5: 245,202-247,884 reverse strand.]

Pelodiscus sinensis (Chinese softshell turtle) (Lost, unsequenced or unnassembled)
[FEN1 Scaffold JH204862.1: 1,045,054-1,050,426 forward strand.]

Anolis carolinensis (Lizard) Scaffold GL343400.1
[PDCD6 Chromosome 6: 54,797,086-54,821,473 forward strand.]
--
REC8 Scaffold GL343400.1: 3,561-17,846 forward strand.
IPO4 Scaffold GL343400.1: 22,954-46,608 reverse strand.
TM9SF1 Scaffold GL343400.1: 53,222-64,987 reverse strand.
--
[FEN1 Scaffold GL343235.1: 1,794,007-1,795,155 forward strand.]

Xenopus tropicalis (African clawed frog) Scaffold GL173075.1
[PDCD6 Scaffold GL172767.1: 2,186,903-2,225,530 reverse strand.]
--
REC8 Scaffold GL173075.1: 516,064-544,485 forward strand.
IPO4 Scaffold GL173075.1: 550,409-585,024 reverse strand.
TM9SF1 Scaffold GL173075.1: 590,924-595,440 reverse strand.
--
[FEN1 Scaffold GL172942.1: 465,710-479,739 reverse strand.]

Ambistoma mexicanum (Axolotl)
No data

Latimeria chalumnae (Coelacanthe) JH128302.1

[PDCD6 Scaffold JH127107.1: 269,519-277,366 reverse strand.]
--
TM9SF1 Scaffold JH128302.1: 1,502-8,220 forward strand.
IPO4 Scaffold JH128302.1: 18,704-103,490 forward strand.
--
[FEN1 Scaffold JH132215.1: 22,719-33,408 reverse strand.]

Danio rerio (Zebrafish) Chromosome 24
TM9SF1 Chromosome 24: 11,662,487-11,680,658 reverse strand.
FEN1 Chromosome 24: 11,681,062-11,691,834 forward strand.
//
PDCD6 Chromosome 24: 12,531,618-12,591,540 reverse strand.
REC8 Chromosome 24: 12,592,132-12,636,552 forward strand.
si:ch211-196f5.2 Chromosome 24: 12,642,817-12,654,471 forward strand.
//
IPO4 Chromosome 24: 12,655,969-12,734,082 reverse strand.
NANOG Chromosome 24: 12,738,148-12,743,225 forward strand.

Gasterosteus aculeatus (Stickleback) groupXXI
FEN1 groupXXI: 3,762,380-3,766,951 reverse strand.
TM9SF1 groupXXI: 3,768,790-3,773,319 forward strand.
NANOG groupXXI: 3,774,464-3,776,625 reverse strand.
IPO4 groupXXI: 3,782,069-3,790,368 forward strand.
ENSGACG00000002283 groupXXI: 3,790,782-3,792,345 reverse strand.
*REC8 groupXXI: 3,812,506-4,072,355 reverse strand. (**Unpredicted, putative**)*
PDCD6 groupXXI: 3,802,461-3,815,446 forward strand.

Xiphophorus maculatus (Platyfish) Scaffold JH556760.1
PDCD6 Scaffold Scaffold JH556760.1: 282,818-299,447 reverse strand.
REC8 Scaffold JH556760.1: 301,923-313,226 forward strand.
ENSXMAG00000006039 Scaffold JH556760.1: 318,942-319,467 forward strand.
IPO4 Scaffold JH556760.1: 319,593-331,269 reverse strand.
NANOG Scaffold JH556760.1: 336,167-338,568 forward strand.
TM9SF1 Scaffold JH556760.1: 340,302-349,658 reverse strand.
FEN1 Scaffold JH556760.1: 350,444-354,084 forward strand.

Tetraodon nigroviridis (Tetraodon) Chromosome 6
FEN1 Chromosome 6: 567,774-571,533 reverse strand.
NANOG Chromosome 6: 574,356-576,008 reverse strand.
IPO4 Chromosome 6: 578,575-591,223 forward strand.

Takifugu rubripes (Fugu) scaffold_95
FEN1 scaffold_95: 953,639-956,818 reverse strand.
TM9SF1 scaffold_95: 958,493-961,878 forward strand.
NANOG scaffold_95: 962,854-965,761 reverse strand.
IPO4 scaffold_95: 967,441-978,129 forward strand.
PDCD6 scaffold_95: 983,720-991,378 forward strand.

Oryzias latipes (Medaka) Chromosome 20
FEN1 Chromosome 20: 10,328,368-10,383,623 reverse strand.
TM9SF1 Chromosome 20: 10,384,025-10,394,637 forward strand.
NANOG Chromosome 20: 10,395,454-10,397,104 reverse strand. ***(Unpredicted, putative)***
IPO4 Chromosome 20: 10,399,577-10,409,031 forward strand.
OLA.21685 Chromosome 20: 10,409,711-10,410,802 reverse strand.
REC8 Chromosome 20: 10,412,370-10,423,341 reverse strand. ***(Unpredicted, putative)***
PDCD6 Chromosome 20: 10,424,970-10,442,516 forward strand.

Gadus morhua (Cod) GeneScaffold_1975
FEN1 GeneScaffold_1975: 8,912-13,062 reverse strand.
TM9SF1 GeneScaffold_1975: 14,471-22,937 forward strand.
*NANOG GeneScaffold_1975: 24,160-26,482 reverse strand. (**Improved prediction**)*
IPO4 GeneScaffold_1975: 29,514-44,374 forward strand.
ENSGMOG00000001847 GeneScaffold_1975: 47,032-48,552 reverse strand.
REC8 GeneScaffold_1975: 54,073-66,092 reverse strand.
PDCD6 GeneScaffold_1975: 66,722-81,943 forward strand.

Sarcopterygian *NANOG* locus (based on Ensembl release 71)

Homo sapiens (Human) Chromosome 12
PEX5 Chromosome 12: 7,341,281-7,371,170 forward strand.
//
APOBEC1 Chromosome 12: 7,801,996-7,818,499 reverse strand.
GDF3 Chromosome 12: 7,842,378-7,848,372 reverse strand.
DPPA3 Chromosome 12: 7,864,050-7,870,155 forward strand.
CLEC4C Chromosome 12: 7,882,011-7,904,201 reverse strand.
NANOGNB Chromosome 12: 7,917,812-7,926,717 forward strand.
NANOG Chromosome 12: 7,940,390-7,948,655 forward strand.
SLC2A14 Chromosome 12: 7,965,108-8,043,744 reverse strand.
NANOGP1 Chromosome 12: 8,025,534-8,052,674 forward strand.
SLC2A3 Chromosome 12: 8,071,826-8,088,871 reverse strand.
FOXJ2 Chromosome 12: 8,185,299-8,208,099 forward strand.
C3AR1 Chromosome 12: 8,210,898-8,219,067 reverse strand.
NECAP1 Chromosome 12: 8,234,807-8,250,367 forward strand.
CLEC4A Chromosome 12: 8,276,228-8,291,203 forward strand.
ZNF705A Chromosome 12: 8,290,733-8,332,642 forward strand.
FAM90A1 Chromosome 12: 8,373,856-8,380,214 reverse strand.
CLEC6A Chromosome 12: 8,608,522-8,630,926 forward strand.
CLEC4D Chromosome 12: 8,662,071-8,674,962 forward strand.
CLEC4E Chromosome 12: 8,685,901-8,693,559 reverse strand.
AICDA Chromosome 12: 8,754,762-8,765,467 reverse strand.
MFAP5 Chromosome 12: 8,789,942-8,815,484 reverse strand.
RIMKLB Chromosome 12: 8,834,196-8,935,691 forward strand.
A2ML1 Chromosome 12: 8,975,068-9,039,597 forward strand.
PHC1 Chromosome 12: 9,066,492-9,094,063 forward strand.
M6PR Chromosome 12: 9,092,959-9,102,551 reverse strand.
KLRG1 Chromosome 12: 9,102,640-9,163,356 forward strand.
A2M Chromosome 12: 9,220,260-9,268,825 reverse strand.
PZP Chromosome 12: 9,301,436-9,360,966 reverse strand.

Tupaia belangeri (Tree Shrew) GeneScaffold_4682
[APOBEC1 GeneScaffold_1365: 777-132,408 reverse strand.]
--
[GDF3 GeneScaffold_5050: 1,990-13,300 reverse strand.]
--
[DPPA3 GeneScaffold_5256: 58,620-61,709 forward strand.]
--
ENSTBEG00000000557 GeneScaffold_4682: 49,136-51,623 forward strand.
(NANOG ortholog)
ENSTBEG00000001034 GeneScaffold_4682: 76,384-107,151 reverse strand.
(SLC2A3/A14 ortholog)
--
[FOXJ2 GeneScaffold_350: 17,537-38,092 forward strand.]
[C3AR1 GeneScaffold_350: 48,244-49,701 reverse strand.]
[NECAP1 GeneScaffold_350: 96,699-110,023 forward strand.]

--
[AICDA GeneScaffold_1364: 12,812-16,728 reverse strand.]
--
[MFAP5 GeneScaffold_3514: 17,545-34,596 reverse strand.]
--
[RIMKLB GeneScaffold_4086: 5,549-30,851 forward strand.]
[A2ML1 GeneScaffold_4086: 77,486-120,462 forward strand.]
--
[PHC1GeneScaffold_1366: 16,687-39,984 forward strand.]
[M6PR GeneScaffold_1366: 42,231-48,603 reverse strand.]
--
[KLRG1 GeneScaffold_2715: 10,295-30,855 forward strand.]
--
[A2M GeneScaffold_4651: 24,611-103,906 reverse strand.]
[PZP GeneScaffold_4651: 144,107-201,475 reverse strand.]
--
[PEX5 GeneScaffold_3826: 66,784-83,606 forward strand.]

Canis lupus familiaris (Dog) Chromosome 27
A2M Chromosome 27: 36,654,072-36,696,052 forward strand.
KLRG1 Chromosome 27: 36,740,984-36,766,777 reverse strand.
M6PR Chromosome 27: 36,786,981-36,796,231 forward strand.
PHC1 Chromosome 27: 36,798,188-36,818,398 reverse strand.
A2ML1 Chromosome 27: 36,844,450-36,883,683 reverse strand.
RIMKLB Chromosome 27: 36,936,000-36,993,347 reverse strand.
MFAP5 Chromosome 27: 37,028,629-37,042,713 forward strand.
AICDA Chromosome 27: 37,064,014-37,073,784 forward strand.
APOBEC1 Chromosome 27: 37,101,330-37,113,663 reverse strand.
GDF3 Chromosome 27: 37,124,095-37,130,390 reverse strand.
DPPA3 Chromosome 27: 37,126,545-37,196,999 forward strand.
NANOGNB Chromosome 27: 37,225,800-37,226,764 forward strand.
ENSCAFG00000013914 Chromosome 27: 37,260,380-37,266,023 forward strand.
(NANOG ortholog)
SLC2A3 Chromosome 27: 37,297,932-37,428,383 reverse strand.
FOXJ2 Chromosome 27: 37,418,553-37,428,174 forward strand.
C3AR1 Chromosome 27: 37,433,792-37,435,246 reverse strand.
NECAP1 Chromosome 27: 37,460,543-37,506,942 forward strand.
CLEC4D Chromosome 27: 37,519,570-37,558,847 forward strand.
CLEC4E Chromosome 27: 37,563,090-37,568,357 reverse strand.
//
PEX5 Chromosome 27: 37,844,434-37,859,930 reverse strand.

Mustela putorius furo (Ferret) GL897020
NANOG Scaffold GL897020.1: 4,084,486-4,088,709 forward strand.
SLC2A3 Scaffold GL897020.1: 4,106,308-4,198,055 reverse strand.
ENSMPUG00000016739 Scaffold GL897020.1: 4,212,258-4,212,933 reverse strand.
(not conserved)
FOXJ2 Scaffold GL897020.1: 4,218,850-4,228,598 forward strand.

C3AR1 Scaffold GL897020.1: 4,233,840-4,235,279 reverse strand.
NECAP1 Scaffold GL897020.1: 4,262,631-4,276,712 forward strand.
CLEC4D Scaffold GL897020.1: 4,345,003-4,354,921 forward strand.
CLEC4E Scaffold GL897020.1: 4,355,790-4,363,783 reverse strand.
//
PEX5 Scaffold GL897020.1: 4,746,404-4,765,590 reverse strand.

[ENSMPUG00000017530 Scaffold GL896932.1: 13,989,045-13,993,832 reverse strand. ***(DPPA3 ortholog)****]*
[GDF3 Scaffold GL896932.1: 14,023,203-14,029,211 forward strand.]
[APOBEC1 Scaffold GL896932.1: 14,038,747-14,049,121 forward strand.]
[AICDA Scaffold GL896932.1: 14,078,698-14,090,953 reverse strand.]
[MFAP5 Scaffold GL896932.1: 14,111,893-14,127,098 reverse strand.]
[RIMKLB Scaffold GL896932.1: 14,157,470-14,216,135 forward strand.]
[A2ML1 Scaffold GL896932.1: 14,283,525-14,307,187 forward strand.]
[PHC1 Scaffold GL896932.1: 14,326,008-14,347,623 forward strand.]
[M6PR Scaffold GL896932.1: 14,350,040-14,357,985 reverse strand.]
[KLRG1 Scaffold GL896932.1: 14,378,032-14,398,600 forward strand.]

Loxodonta africana (Elephant) SuperContig scaffold_15
PEX5 SuperContig scaffold_15: 52,009,254-52,029,451 forward strand.
//
CLEC4E SuperContig scaffold_15: 52,569,860-52,575,411 forward strand.
CLEC4D SuperContig scaffold_15: 52,609,712-52,616,811 reverse strand.
ENSLAFG00000029174 SuperContig scaffold_15: 52,753,478-52,755,563 reverse strand. ***(CLEC4A paralog)***
ENSLAFG00000010246 SuperContig scaffold_15: 52,818,186-52,830,999 reverse strand. ***(CLEC4A paralog)***
ENSLAFG00000022886 SuperContig scaffold_15: 52,931,714-52,933,991 reverse strand. ***(CLEC4A paralog)***
NECAP1 SuperContig scaffold_15: 53,199,520-53,210,509 reverse strand.
C3AR1 SuperContig scaffold_15: 53,239,140-53,240,621 forward strand.
FOXJ2 SuperContig scaffold_15: 53,246,025-53,257,497 reverse strand.
ENSLAFG00000004432 SuperContig scaffold_15: 53,383,596-53,395,605 forward strand. ***(SLC2A3/A14 ortholog)***
ENSLAFG00000012934 SuperContig scaffold_15: 53,444,918-53,449,716 reverse strand. ***(NANOG ortholog)***
LOC100655545 SuperContig scaffold_15: 53,476,159-53,500,682 reverse strand. ***(Putative NANOGNB ortholog, from ncbi)***
CLEC4C SuperContig scaffold_15: 53,535,532-53,542,326 forward strand.
GDF3 SuperContig scaffold_15: 53,722,764-53,727,873 forward strand.
APOBEC1 SuperContig scaffold_15: 53,748,002-53,749,555 forward strand.
AICDA SuperContig scaffold_15: 53,777,928-53,780,982 reverse strand.
MFAP5 SuperContig scaffold_15: 53,812,592-53,823,364 reverse strand.
RIMKLB SuperContig scaffold_15: 53,901,418-53,932,897 forward strand.
A2ML1 SuperContig scaffold_15: 53,998,389-54,039,712 forward strand.
PHC1 SuperContig scaffold_15: 54,085,662-54,111,313 forward strand.
M6PR SuperContig scaffold_15: 54,113,666-54,117,969 reverse strand.

A2M SuperContig scaffold_15: 54,204,907-54,252,414 reverse strand.
ENSLAFG00000028486 SuperContig scaffold_15: 54,345,293-54,421,781 reverse strand. ***(A2M/PZP paralog)***
ENSLAFG00000002095 SuperContig scaffold_15: 54,436,023-54,497,916 reverse strand. ***(A2M/PZP paralog)***
ENSLAFG00000003324 SuperContig scaffold_15: 54,522,254-54,567,186 reverse strand. ***(A2M/PZP paralog)***

Bos taurus (Cow) Chromosome 5
ENSBTAG00000038461 Chromosome 5: 101,210,292-101,266,954 forward strand. ***(Possible PZP ortholog)***
A2M Chromosome 5: 101,298,127-101,346,611 forward strand.
KLRG1 Chromosome 5: 101,402,262-101,419,313 reverse strand.
M6PR Chromosome 5: 101,439,877-101,452,407 forward strand.
PHC1 Chromosome 5: 101,453,054-101,472,170 reverse strand.
A2ML1 Chromosome 5: 101,495,664-101,545,688 reverse strand.
RIMKLB Chromosome 5: 101,581,511-101,618,950 reverse strand.
MFAP5 Chromosome 5: 101,647,629-101,659,377 forward strand.
BT.56893 Chromosome 5: 101,686,492-101,690,821 forward strand. ***(AICDA ortholog)***
APOBEC1 Chromosome 5: 101,725,366-101,730,873 reverse strand.
GDF3 Chromosome 5: 101,751,209-101,757,820 reverse strand.
STELLA Chromosome 5: 101,791,824-101,799,616 forward strand. ***(DDPA3 ortholog)***
NANOG Chromosome 5: 101,871,073-101,876,226 forward strand.
SLC2A3 Chromosome 5: 101,896,622-101,909,500 reverse strand.
FOXJ2 Chromosome 5: 102,003,895-102,023,009 forward strand.
C3AR1 Chromosome 5: 102,025,881-102,035,208 reverse strand.
CLEC4A Chromosome 5: 102,076,231-102,089,108 forward strand.
CLEC6A Chromosome 5: 102,101,961-102,118,594 forward strand.
CLEC4D Chromosome 5: 102,144,410-102,152,592 forward strand.
CLEC4E Chromosome 5: 102,158,068-102,168,602 reverse strand.
//
PEX5 Chromosome 5: 103,530,546-103,547,919 reverse strand.

Sus Scrofa (Pig) Chromosome 1 and chromosome 5 (n.b:
ENSSSCG00000021112 Chromosome 1: 187,485,435-187,623,277 reverse strand. ***(THSD4 paralog)***
NANOG Chromosome 1: 187,711,367-187,712,525 forward strand. ***(intronless retropseudogene)***
THSD4 Chromosome 1: 187,774,212-188,055,229 forward strand.
--
[KLRB1 Chromosome 5: 65,081,358-65,094,180 forward strand.]
[ENSSSCG00000000659 Chromosome 5: 65,110,350-65,162,280 forward strand. ***(unclear similar to A2M?)****]*
[ENSSSCG00000024402 Chromosome 5: 65,186,983-65,233,549 forward strand. ***(A2M/PZP ortholog)****]*
[A2M Chromosome 5: 65,243,776-65,320,386 forward strand.]

[PHC1 Chromosome 5: 65,355,428-65,377,762 forward strand.]
[M6PR Chromosome 5: 65,378,672-65,385,109 reverse strand.]
[KLRG1 Chromosome 5: 65,412,927-65,430,937 forward strand.]
[A2ML1 Chromosome 5: 65,445,195-65,479,118 reverse strand.]
[RIMKLB Chromosome 5: 65,517,873-65,546,224 reverse strand.]
[MFAP5 Chromosome 5: 65,575,791-65,589,682 forward strand.]
[AICDA Chromosome 5: 65,607,546-65,616,194 forward strand.]
[APOBEC1 Chromosome 5: 65,642,441-65,650,313 reverse strand.]
[GDF3 Chromosome 5: 65,666,119-65,671,845 reverse strand.]
[SLC2A3 Chromosome 5: 65,800,934-65,810,886 forward strand.]
--
[C3AR1 Scaffold GL896371.1: 25,480-26,910 forward strand.]
[FOXJ2 Scaffold GL896371.1: 34,712-37,223 reverse strand.]

Pterobat vampirus (Megabat) scaffold_8071
[PZP GeneScaffold_1346: 10,062-38,883 reverse strand.]
--
[A2M GeneScaffold_3212: 17,447-56,594 reverse strand.]
--
[AICDA GeneScaffold_927: 17,578-24,633 reverse strand.]
[MFAP5 GeneScaffold_927: 39,657-49,075 reverse strand.
[RIMKLB GeneScaffold_927: 81,537-94,716 forward strand.]
[A2ML1 GeneScaffold_927: 123,632-154,266 forward strand.]
[PHC1 GeneScaffold_927: 182,831-199,019 forward strand.]
[M6PR GeneScaffold_927: 201,356-205,936 reverse strand.]
[KLRG1 GeneScaffold_927: 222,062-227,668 forward strand.]
--
[NECAP1 scaffold_12289: 174-8,772 forward strand.]
[CLEC4A scaffold_12289: 25,263-36,562 forward strand.]
--
[FOXJ2 scaffold_12151: 8,802-20,063 forward strand.]
[C3AR1 scaffold_12151: 24,658-26,109 reverse strand.]
--
ENSPVAG00000006989 scaffold_8071: 64,939-68,863 forward strand. ***(NANOG ortholog)***
DPPA3 scaffold_8071: 14,014-17,346 forward strand.
--
[GDF3 GeneScaffold_928: 15,886-21,263 reverse strand.]
[APOBEC1 GeneScaffold_928: 2,672-11,501 reverse strand.]

Myotis lucifugus (Microbat) Scaffold GL429777
[ENSMLUG00000029325 Scaffold GL431244: 34,777-40,205 reverse strand. ***(Nanog paralog)****]*
--
CLEC4E Scaffold GL429777: 8,719,254-8,724,505 forward strand.
CLEC4D Scaffold GL429777: 8,730,741-8,736,802 reverse strand.
CLEC6A Scaffold GL429777: 8,759,551-8,770,934 reverse strand.
CLEC4A Scaffold GL429777: 8,803,868-8,827,619 reverse strand.

NECAP1 Scaffold GL429777: 8,851,070-8,861,864 reverse strand.
C3AR1 Scaffold GL429777: 8,889,334-8,890,770 forward strand.
FOXJ2 Scaffold GL429777: 8,896,497-8,906,478 reverse strand.
ENSMLUG00000026617 Scaffold GL429777: 8,924,555-8,930,089 forward strand.
(CLK3 paralog)
ENSMLUG00000004862 Scaffold GL429777: 8,999,385-9,009,389 forward strand.
(SLC2A3/A14 ortholog)
ENSMLUG00000030580 Scaffold GL429777: 9,038,390-9,043,290 forward strand.
(NANOG ortholog)
ENSMLUG00000030648 Scaffold GL429777: 9,138,539-9,141,019 reverse strand.
(putative PLRG1 paralog)
GDF3 Scaffold GL429777: 9,155,237-9,160,384 forward strand.
ENSMLUG00000012233 Scaffold GL429777: 9,193,256-9,194,508 forward strand.
(putative EIF4A3 paralog)
AICDA Scaffold GL429777: 9,201,968-9,205,318 reverse strand.
MFAP5 Scaffold GL429777: 9,224,380-9,234,907 reverse strand.
RIMKLB Scaffold GL429777: 9,292,923-9,302,722 forward strand.
A2ML1 Scaffold GL429777: 9,329,741-9,363,915 forward strand.
PHC1 Scaffold GL429777: 9,388,453-9,406,492 forward strand.
M6PR Scaffold GL429777: 9,408,708-9,414,709 reverse strand.
ENSMLUG00000023713 Scaffold GL429777: 9,429,948-9,451,415 reverse strand.
(A2M paralog)
ENSMLUG00000023672 Scaffold GL429777: 9,488,462-9,489,622 reverse strand.
(ADIPOR2 paralog)
ENSMLUG00000011177 Scaffold GL429777: 9,518,526-9,561,356 reverse strand.
(A2M paralog)
PZP Scaffold GL429777: 9,585,876-9,631,546 reverse strand.

Dipodomys ordii (Kangaroo rat) unassembled or lost region
[Pex5 GeneScaffold_3293: 672-8,464 forward strand.]
--
[M6pr GeneScaffold_1752: 15,257-17,759 reverse strand.]
--
[Phc1 GeneScaffold_1752: 493-11,290 forward strand.]
--
[Rimklb GeneScaffold_4981: 15,156-20,917 forward strand.]
--
[C3ar1 scaffold_54052: 353-1,480 reverse strand.]
--
[FoxJ2 GeneScaffold_445: 448-7,327]

Oryctolagus cuniculus (Rabbit) Chromosome 8
PEX5 Chromosome 8: 33,168,761-33,188,224 forward strand.
//
CLEC4E Chromosome 8: 33,490,792-33,496,527 forward strand.
CLEC4D Chromosome 8: 33,502,476-33,507,857 reverse strand.
CLEC4A Chromosome 8: 33,544,270-33,562,662 reverse strand.
NECAP1 Chromosome 8: 33,589,781-33,603,760 reverse strand.

C3AR1 Chromosome 8: 33,623,688-33,625,130 forward strand.
FOXJ2 Chromosome 8: 33,631,674-33,642,273 reverse strand.
ENSOCUG00000008575 Chromosome 8: 33,740,676-33,752,089 forward strand. ***(SLC2A3/A14 ortholog)***
ENSOCUG00000013783 Chromosome 8: 33,789,671-33,797,889 reverse strand. ***(NANOG ortholog)***
STELLA Chromosome 8: 33,836,113-33,860,501 reverse strand ***(Unpredicted, putative)***
LAMR1 Chromosome 8: 33,878,390-33,879,607 reverse strand.
GDF3 Chromosome 8: 33,883,835-33,892,453 forward strand.
APOBEC1 Chromosome 8: 33,903,808-33,914,877 forward strand.
AICDA Chromosome 8: 33,935,192-33,939,710 reverse strand.
MFAP5 Chromosome 8: 33,976,095-33,991,847 reverse strand.
RIMKLB Chromosome 8: 34,042,576-34,075,994 forward strand.
A2ML1 Chromosome 8: 34,127,102-34,166,544 forward strand.
PHC1 Chromosome 8: 34,208,871-34,232,745 forward strand.
M6PR Chromosome 8: 34,234,648-34,240,119 reverse strand.
KLRG1 Chromosome 8: 34,255,768-34,267,255 forward strand.
//
ENSOCUG00000000313 Chromosome 8: 34,453,884-34,506,874 forward strand. ***(putative A2M ortholog)***
ENSOCUG00000027871 Chromosome 8: 34,527,366-34,592,966 forward strand. ***(putative PZP ortholog)***
A2M Chromosome 8: 34,619,727-34,681,527 forward strand.
PZP Chromosome 8: 34,900,682-34,956,253 forward strand.

Mus musculus (Mouse) Chromosome 6
A2M Chromosome 6: 121,636,173-121,679,237 forward strand.
//
Klrg1 Chromosome 6: 122,270,596-122,282,882 reverse strand.
M6pr Chromosome 6: 122,308,720-122,317,680 forward strand.
Phc1 Chromosome 6: 122,317,731-122,340,561 reverse strand.
1700063H04Rik Chromosome 6: 122,391,379-122,392,687 forward strand. ***(no known ortholog)***
Rimklb Chromosome 6: 122,447,296-122,486,313 reverse strand.
Mfap5 Chromosome 6: 122,505,845-122,529,290 forward strand.
Aicda Chromosome 6: 122,553,801-122,564,180 forward strand.
Apobec1 Chromosome 6: 122,577,792-122,602,444 reverse strand.
Gdf3 Chromosome 6: 122,605,403-122,610,087 reverse strand.
Dppa3 Chromosome 6: 122,626,410-122,630,272 forward strand.
Nanog Chromosome 6: 122,707,489-122,714,633 forward strand.
Slc2a3 Chromosome 6: 122,727,809-122,801,640 reverse strand.
Foxj2 Chromosome 6: 122,819,914-122,845,366 forward strand.
C3ar1 Chromosome 6: 122,847,138-122,856,161 reverse strand.
Necap1 Chromosome 6: 122,874,557-122,888,934 forward strand.
Gm5316 Chromosome 6: 122,900,136-122,900,405 reverse strand.
Clec4a1 Chromosome 6: 122,921,848-122,934,619 forward strand.
Clec4a3 Chromosome 6: 122,952,515-122,969,875 forward strand.

Clec4a4 Chromosome 6: 122,990,367-123,024,105 forward strand.
Clec4b1 Chromosome 6: 123,049,962-123,071,555 forward strand.
Clec4a2 Chromosome 6: 123,106,428-123,143,999 forward strand.
Clec4b2 Chromosome 6: 123,172,893-123,204,671 forward strand.
Clec4n Chromosome 6: 123,229,843-123,247,021 forward strand. ***(CLEC6A ortholog)***
Clec4d Chromosome 6: 123,262,107-123,275,268 forward strand.
Clec4e Chromosome 6: 123,281,789-123,289,870 reverse strand.
//
Pex5 Chromosome 6: 124,396,816-124,415,067 reverse strand.
//
Pzp Chromosome 6: 128,483,567-128,526,720 reverse strand.

Cavia porcellus (Guinea pig) scaffold_28
PZP scaffold_28: 1,249,735-1,334,334 reverse strand.
A2M scaffold_28: 1,402,401-1,450,979 forward strand.
KLRG1 scaffold_28: 1,591,925-1,600,780 reverse strand.
M6PR scaffold_28: 1,636,679-1,644,064 forward strand.
PHC1 scaffold_28: 1,646,397-1,664,744 reverse strand.
RIMKLB scaffold_28: 1,812,157-1,848,279 reverse strand.
ENSCPOG00000019389 scaffold_28: 1,937,809-1,940,598 forward strand. ***(AICDA ortholog)***
ENSCPOG00000015181 scaffold_28: 1,973,006-1,979,855 reverse strand. ***(SLC2A3/A14 ortholog)***
ENSCPOG00000027331 scaffold_28: 2,008,744-2,012,576 reverse strand. ***(APOBEC1 ortholog)***
ENSCPOG00000001939 scaffold_28: 2,032,194-2,035,065 reverse strand. ***(GDF3 ortholog)***
ENSCPOG00000025925 scaffold_28: 2,123,076-2,124,507 reverse strand. ***(Kin1-like)***
ENSCPOG00000008888 scaffold_28: 2,167,077-2,172,849 forward strand. ***(NANOG paralog)***
ENSCPOG00000025468 scaffold_28: 2,238,462-2,245,039 forward strand. ***(MFAP5 ortholog)***
ENSCPOG00000020399 scaffold_28: 2,263,346-2,266,156 forward strand. ***(AICDA ortholog)***
ENSCPOG00000003074 scaffold_28: 2,311,347-2,329,400 forward strand. ***(SLC2A3/SLC2A14 ortholog)***
ENSCPOG00000023433 scaffold_28: 2,362,465-2,365,223 reverse strand. ***(AICDA ortholog)***
ENSCPOG00000003749 scaffold_28: 2,389,842-2,401,005 reverse strand. ***(MFAP5 ortholog)***
ENSCPOG00000002621 scaffold_28: 2,498,785-2,504,514 reverse strand. ***(NANOG paralog)***
ENSCPOG00000025421 scaffold_28: 2,550,991-2,552,041 forward strand. ***(Kin1-like)***
ENSCPOG00000020509 scaffold_28: 2,577,374-2,586,299 forward strand. ***(CLEC4C ortholog)***

ENSCPOG00000015516 scaffold_28: 2,609,659-2,612,542 forward strand. ***(GDF3 ortholog)***
ENSCPOG00000020166 scaffold_28: 2,632,503-2,636,530 forward strand.
(APOBEC1 ortholog)
ENSCPOG00000011295 scaffold_28: 2,677,254-2,687,378 reverse strand.
(SLC2A3/A14 ortholog)
RPL19 scaffold_28: 2,726,604-2,726,756 forward strand.
FOXJ2 scaffold_28: 2,780,590-2,790,344 forward strand.
C3AR1 scaffold_28: 2,794,006-2,801,130 reverse strand.
NECAP1 scaffold_28: 2,831,283-2,842,961 forward strand.
ENSCPOG00000005961 scaffold_28: 2,888,427-2,900,152 forward strand.
(CLEC4A ortholog)

Ictidomys tridecemlineatus (Squirrel) JH393702.1
[GDF3 Scaffold JH394656.1: 7,456-16,783 forward strand.]
--
[APOBEC1 Scaffold JH396108.1: 628-6,208 reverse strand.]
--
[AICDA Scaffold JH393680.1: 25,039-28,617 reverse strand.]
[MFAP5 Scaffold JH393680.1: 58,189-71,325 reverse strand.]
[RIMKLB Scaffold JH393680.1: 127,261-164,427 forward strand.]
[PHC1 Scaffold JH393680.1: 277,438-299,846 forward strand.]
[M6PR Scaffold JH393680.1: 302,166-307,134 reverse strand.]
[KLRG1 Scaffold JH393680.1: 339,526-352,620 forward strand.]
[A2M Scaffold JH393680.1: 424,602-468,167 reverse strand.]
[PZP Scaffold JH393680.1: 497,137-542,364 reverse strand.]
--
ENSSTOG00000022241 Scaffold JH393702.1: 181,813-187,691 forward strand.
(NANOG ortholog)
ENSSTOG00000003250 Scaffold JH393702.1: 224,259-234,288 reverse strand.
(SLC2A3/A14 ortholog)
FOXJ2 Scaffold JH393702.1: 324,790-341,932 forward strand.
C3AR1 Scaffold JH393702.1: 347,842-349,296 reverse strand.
NECAP1 Scaffold JH393702.1: 372,787-383,582 forward strand.

Macropus eugenii (Wallaby) Scaffold1128 (region unassembled)
[NECAP1 GeneScaffold_1326: 433-6,677 forward strand.]
--
[FOXJ2 Scaffold36584: 3,203-18,512 forward strand.]
--
NANOG Scaffold1128: 11737:14400 reverse strand ***(Unpredicted, putative)***
--
[M6PR Scaffold8651: 20,159-23,685 reverse strand.]
--
[ENSMEUG00000004863 GeneScaffold_8348: 3,328-98,946 reverse strand.] ***(A2M ortholog)***
--
[PHC1 Scaffold8651: 941-17,534 forward strand.]

--
[RIMKLB GeneScaffold_7415: 403-22,915 forward strand.]
--
[MFAP5 GeneScaffold_9558: 5,280-21,213 reverse strand._]
--
[AICDA Scaffold22685: 14,800-21,635 forward strand.]
--
[APOBEC1 Scaffold218: 3,902-13,179 reverse strand.]
--
[GDF3 Scaffold218: 31,996-38,968 reverse strand.]

Sarcophilus harrisii (Tasmanian devil) Scaffold GL861820.1 and GL840998.1
[NECAP1 Scaffold GL861610.1: 2,477,062-2,487,122 reverse strand.]
[C3AR1 Scaffold GL861610.1: 2,523,561-2,525,066 forward strand.]
[FOXJ2 Scaffold GL861610.1: 2,531,131-2,559,973 reverse strand.]
--
NANOG Scaffold GL840998.1:1908-2304 reverse strand. ***(Unpredicted, putative)***
--
ENSSHAG00000003164 Scaffold GL861820.1: 53,915-65,835 forward strand.
(SLC2A3/A14 ortholog)
ENSSHAG00000004362 Scaffold GL861820.1: 100,942-104,884 reverse strand.
(NANOG ortholog)
--
[ENSSHAG00000002556 Scaffold GL861611.1: 35,413-107,589 forward strand.
***(A2M ortholog)**]*
[M6PR Scaffold GL861611.1: 144,058-147,431 forward strand.]
[PHC1 Scaffold GL861611.1: 149,764-164,137 reverse strand.]
[RIMKLB Scaffold GL861611.1: 259,962-345,176 reverse strand.]
[MFAP5 Scaffold GL861611.1: 396,395-416,119 forward strand.]
[AICDA Scaffold GL861611.1: 453,160-461,699 forward strand.]
[APOBEC1 Scaffold GL861611.1: 481,548-486,863 reverse strand.]
[GDF3 Scaffold GL861611.1: 518,092-525,141 reverse strand.]

Monodelphis domestica (Opossum) Chromosome 8
NANOGPS Chromosome 8: 44,916,393-44-916-752 reverse strand ***(unpredicted putative NANOG pseudogene)***
//
NECAP1 Chromosome 8: 104,396,902-104,408,000 reverse strand.
FOXJ2 Chromosome 8: 104,446,786-104,483,051 reverse strand.
ENSMODG00000017976 Chromosome 8: 104,571,681-104,583,767 forward strand.
(SLC2A3/A14 ortholog)
ENSMODG00000017979 Chromosome 8: 104,628,273-104,648,421 reverse strand.
(NANOG ortholog)
ENSMODG00000017988 Chromosome 8: 104,682,602-104,771,610 forward strand.
(A2M ortholog)
M6PR Chromosome 8: 104,814,941-104,821,045 forward strand.
PHC1 Chromosome 8: 104,819,445-104,838,359 reverse strand.
RIMKLB Chromosome 8: 104,932,756-105,011,275 reverse strand.

MFAP5 Chromosome 8: 105,106,376-105,130,618 forward strand.
AICDA Chromosome 8: 105,169,817-105,176,826 forward strand.
APOBEC1 Chromosome 8: 105,213,203-105,222,916 reverse strand.
GDF3 Chromosome 8: 105,231,539-105,243,167 reverse strand.
//
PEX5 Chromosome 8: 108,748,713-108,782,486 forward strand.

Ornithorhynchus anatinus (Platypus) SuperContig Contig7532 and SuperContig Contig6405
AICDA SuperContig Contig7532: 8,020-10,175 forward strand.
APOBEC1 SuperContig Contig7532: 17,916-19,361 reverse strand.
NANOG SuperContig Contig7532: 8,020-10,175 forward strand.
--
NANOG SuperContig Contig6405: 9,675-18,937 forward strand. ***(Unpredicted, putative)***
GDF3 SuperContig Contig6405: 14,028-14,362 forward strand. ***(Unpredicted, putative)***
ENSOANG00000015730 SuperContig Contig6405: 26,113-31,518 reverse strand. ***(SLC2A3/A14 ortholog)***
ENSOANG00000028775 SuperContig Contig6405: 40,503-41,705 forward strand. ***(CLEC4A/B/C/D/6A ortholog)***

Taeniopygia guttata (Zebrafinch) Chromosome 1
PEX5 Chromosome 12: 7,341,281-7,371,170 forward strand.
//
ENSTGUG00000018189 Chromosome 1: 87,761,449-87,789,588 forward strand. ***(A2M-like)***
ENSTGUG00000013069 Chromosome 1: 87,799,023-87,823,789 forward strand. ***(A2M-like)***
ENSTGUG00000013071 Chromosome 1: 87,832,309-87,868,297 forward strand. ***(A2M/PZP paralog)***
ENSTGUG00000013087 Chromosome 1: 87,871,735-87,901,436 reverse strand. ***(A2M/PZP paralog)***
M6PR Chromosome 1: 87,919,346-87,921,715 forward strand.
ENSTGUG00000013100 Chromosome 1: 87,925,668-87,937,652 reverse strand. ***(PHC1 ortholog)***
ENSTGUG00000018526 Chromosome 1: 87,984,303-88,005,294 forward strand. ***(A2ML1 paralog)***
ENSTGUG00000013105 Chromosome 1: 88,014,982-88,053,990 reverse strand. ***(A2ML1 paralog)***
ENSTGUG00000013107 Chromosome 1: 88,034,247-88,046,764 reverse strand. ***(A2ML1 paralog)***
RIMKLB Chromosome 1: 88,087,078-88,118,910 reverse strand.
ENSTGUG00000013114 Chromosome 1: 88,149,696-88,151,431 forward strand. ***(MFAP5 ortholog)***
AICDA Chromosome 1: 88,165,765-88,167,287 forward strand.
APOBEC1 Chromosome 1: 88,175,757-88,177,346 reverse strand.
ENSTGUG00000013120 Chromosome 1: 88,188,702-88,189,455 reverse strand.

(APOBEC1 paralog)
ENSTGUG00000013121 Chromosome 1: 88,224,132-88,226,482 forward strand.
(NANOG paralog)
ENSTGUG00000013123 Chromosome 1: 88,232,783-88,235,011 reverse strand.
(NANOG paralog)
ENSTGUG00000013125 Chromosome 1: 88,260,212-88,266,209 reverse strand.
(SLC2A3/SLCA14 ortholog)
FOXJ2 Chromosome 1: 88,321,169-88,329,070 forward strand.
C3AR1 Chromosome 1: 88,332,888-88,333,809 reverse strand.
NECAP1 Chromosome 1: 88,341,270-88,345,749 forward strand.
ENSTGUG00000013141 Chromosome 1: 88,353,695-88,384,211 forward strand.
(CLEC4A/B/C/D/E paralog)
ENSTGUG00000018267 Chromosome 1: 88,371,369-88,376,157 forward strand.
(CLEC4A/B/C/D/E paralog)
ENSTGUG00000013144 Chromosome 1: 88,392,687-88,393,505 forward strand.
(CLEC4A/B/C/D/E paralog)

Meleagris gallopavo (Turkey) Chromosome 1
NECAP1 Chromosome 1: 79,216,235-79,222,252 reverse strand.
FOXJ2 Chromosome 1: 79,234,113-79,243,590 reverse strand.
ENSMGAG00000013938 Chromosome 1: 79,299,674-79,305,166 forward strand.
(SLC2A3/SLC2A14 ortholog)
ENSMGAG00000016732 Chromosome 1: 79,347,037-79,348,940 forward strand.
(NANOG paralog)
ENSMGAG00000013941 Chromosome 1: 79,355,894-79,358,232 reverse strand.
(NANOG paralog)
ENSMGAG00000013931 Chromosome 1: 79,181,355-79,192,158 forward strand.
(FCBGP ortholog)
AICDA Chromosome 1: 79,433,286-79,434,812 reverse strand.
MFAP5 Chromosome 1: 79,449,126-79,460,201 reverse strand.
A2ML1 Chromosome 1: 79,614,577-79,639,918 forward strand.
PHC1 Chromosome 1: 79,757,698-79,772,322 forward strand.
M6PR Chromosome 1: 79,775,330-79,777,751 reverse strand.
ENSMGAG00000013956 Chromosome 1: 79,801,005-79,817,143 forward strand.
(A2M/PZP paralog)
A2M Chromosome 1: 79,843,315-79,953,827 reverse strand.
ENSMGAG00000013976 Chromosome 1: 79,964,255-80,008,796 reverse strand.
(A2M-like)
//
PEX5 Chromosome 1: 81,091,977-81,102,163 forward strand.

Gallus gallus (Chick) Chromosome 1
NECAP1 Chromosome 1: 75,222,848-75,230,396 reverse strand.
FOXJ2 Chromosome 1: 75,249,235-75,287,526 reverse strand.
SLC2A3 Chromosome 1: 75,323,723-75,331,877 forward strand.
ENSGALG00000028398 Chromosome 1: 75,344,339-75,363,976 forward strand.
(NANOG paralog)
NANOG Chromosome 1: 75,366,745-75,371,263 reverse strand.

AICDA Chromosome 1: 75,409,240-75,413,213 reverse strand.
MFAP5 Chromosome 1: 75,420,699-75,436,042 reverse strand.
RIMKLB Chromosome 1: 75,460,567-75,494,768 forward strand.
A2ML1 Chromosome 1: 75,534,046-75,551,354 forward strand.
ENSGALG00000014252 Chromosome 1: 75,569,495-75,630,018 forward strand. ***(A2ML1 paralog)***
PHC1 Chromosome 1: 75,640,948-75,660,086 forward strand.
GGA.4354 Chromosome 1: 75,660,141-75,665,386 reverse strand. ***(M6PR ortholog)***
ENSGALG00000022964 Chromosome 1: 75,682,822-75,711,590 forward strand. ***(A2M/PZP paralog)***
ENSGALG00000011926 Chromosome 1: 75,713,572-75,750,893 reverse strand. ***(A2M/PZP paralog)***
ENSGALG00000022962 Chromosome 1: 75,769,353-75,804,565 reverse strand. ***(A2M-like)***
ENSGALG00000011930 Chromosome 1: 75,817,331-75,854,119 reverse strand. ***(A2M-like)***
//
PEX5 Chromosome 1: 76,914,600-76,923,832 forward strand.

Pelodiscus sinensis (Chinese softshell turtle) Scaffold JH207983
NECAP1 Scaffold JH207983.1: 12,377-17,567 reverse strand.
C3AR1 Scaffold JH207983.1: 40,785-42,254 forward strand.
FOXJ2 Scaffold JH207983.1: 50,790-83,889 reverse strand.
ENSPSIG00000000059 Scaffold JH207983.1: 158,889-159,374 reverse strand. ***(not conserved)***
ENSPSIG00000011013 Scaffold JH207983.1: 168,898-184,356 forward strand. ***(SLC2A3/A14 ortholog)***
ENSPSIG00000012067 Scaffold JH207983.1: 219,310-230,273 forward strand. ***(NANOG paralog)***
ENSPSIG00000012334 Scaffold JH207983.1: 235,450-240,970 reverse strand. ***(NANOG paralog)***
ENSPSIG00000012568 Scaffold JH207983.1: 298,547-300,744 reverse strand. ***(APOBEC1 ortholog)***
ENSPSIG00000012769 Scaffold JH207983.1: 307,476-309,212 forward strand. ***(GTF2IRD ortholog)***
ENSPSIG00000012791 Scaffold JH207983.1: 318,254-337,898 forward strand. ***(APOBEC1 ortholog)***
AICDA Scaffold JH207983.1: 402,749-404,077 reverse strand.
MFAP5 Scaffold JH207983.1: 426,297-447,877 reverse strand.
RIMKLB Scaffold JH207983.1: 485,337-557,951 forward strand.
A2ML1 Scaffold JH207983.1: 426,297-447,877 forward strand.

Anolis carolinensis (Lizard) Chromosome 2
[NECAP1 Scaffold GL343236.1: 1,163,113-1,186,590 reverse strand.]
[C3AR1 Scaffold GL343236.1: 1,218,277-1,219,686 forward strand.]
[FOXJ2 Scaffold GL343236.1: 1,228,527-1,248,334 reverse strand.]
[ENSACAG00000011337 Scaffold GL343236.1: 1,391,723-1,422,178 forward

strand. ***(SLC2A3/A14 ortholog)****]*
--
YIPF2 Chromosome 2: 80,878,265-80,894,452 reverse strand.
ENSACAG00000015317 Chromosome 2: 80,930,150-80,939,167 reverse strand. ***(NANOG ortholog)***
ENSACAG00000015139 Chromosome 2: 80,967,859-81,091,650 forward strand. ***(A2ML1 paralog)***
ABOBEC1 Chromosome 2: 81,083,205-81,097,885 reverse strand.
ENSACAG00000015126 Chromosome 2: 81,152,127-81,252,023 reverse strand. ***(A2ML1 paralog)***
RIMKLB Chromosome 2: 81,329,232-81,364,300 reverse strand.
MFAP5 Chromosome 2: 81,436,029-81,459,930 forward strand.
AICDA Chromosome 2: 81,518,110-81,535,131 forward strand.
PHC1 Chromosome 2: 81,549,095-81,590,307 forward strand.
M6PR Chromosome 2: 81,594,900-81,601,337 reverse strand.

Xenopus tropicalis (African clawed frog) Scaffold GL173371
aicda Scaffold GL173371.1: 30,653-32,541 forward strand.
slc2a3 Scaffold GL173371.1: 35,087-41,613 reverse strand.
ENSXETG00000023904 Scaffold GL173371.1: 60,614-84,366 forward strand. ***(NUMA1/CNTRL like)***
foxj2 Scaffold GL173371.1: 122,219-132,362 reverse strand.
c12orf57 Scaffold GL173371.1: 136,171-137,145 forward strand.
c3ar1 Scaffold GL173371.1: 140,348-141,442 reverse strand.
pex5 Scaffold GL173371.1: 158,861-170,205 forward strand.

Ambistoma mexicanum (Axolotl) LG3 (physical map no genomic coordinates)
NANOG LG3
C3AR1 LG3

Latimeria chalumnae (Coelacanthe) Scaffold JH127875.1
ENSLACG00000007520 Scaffold JH127875.1: 281,918-301,641 forward strand. ***(SLC2A3/SLC2A14 ortholog)***
NANOG2 Scaffold JH127875.1: 316,921-323,744 reverse strand. ***(Unpredicted, putative)***
NANOG1 Scaffold JH127875.1: 345,415-352,530 reverse strand. ***(Unpredicted, putative)***
AICDA Scaffold JH127875.1: 411,538-412,095 reverse strand.
ENSLACG00000009356 Scaffold JH127875.1: 414,295-417,089 forward strand. ***(not conserved)***
FOXJ2 Scaffold JH127875.1: 468,273-471,065 reverse strand.
RIMKLB Scaffold JH127875.1: 516,942-550,210 reverse strand.
MFAP5 Scaffold JH127875.1: 610,532-622,661 forward strand.
ENSLACG00000011705 JH127875.1: 627,887-655,437 reverse strand. ***(NUMA1/CNTRL like)***

Danio rerio (Zebrafish) Chromosome 16 and Chromosome 19
pex5 Chromosome 16: 13,812,635-13,847,401 reverse strand.

//
CU694256.1 Chromosome 16: 14,228,375-14,238,716 reverse strand.
(SLC2A3/SLC2A14 ortholog)
//
aicda Chromosome 16: 14,411,898-14,417,073 forward strand.
mfap5 Chromosome 16: 14,421,237-14,440,378 reverse strand.
zgc:172086 Chromosome 16: 14,462,325-14,475,159 reverse strand. ***(SBK2 ortholog)***
epn1 Chromosome 16: 14,481,759-14,507,949 forward strand.
foxj2 Chromosome 16: 14,509,915-14,538,450 reverse strand.
//
m6pr Chromosome 16: 19,641,611-19,648,583 reverse strand.
//
phc1 Chromosome 16: 34,257,008-34,274,819 forward strand.
--
slc2a3a Chromosome 19: 10,307,750-10,335,066 reverse strand.
//
necap1 Chromosome 19: 11,018,578-11,030,565 forward strand.

Gasterosteus aculeatus (Stickleback) groupXX and scaffold_149
PEX5 groupXX: 11,581,081-11,589,204 reverse strand.
PHC1.2 groupXX: 11,905,700-11,909,566 reverse strand.
M6PR groupXX: 11,911,904-11,914,978 reverse strand.
//
ENSGACG00000010483 groupXX: 11,997,359-12,005,729 reverse strand.
(SLC2A3/SLC2A14 ortholog)
//
AICDA groupXX: 12,050,972-12,052,426 forward strand.
NECAP1 groupXX: 12,053,199-12,058,279 forward strand.
EPN1 groupXX: 12,077,130-12,083,862 forward strand.
ENSGACG00000010572 groupXX: 12,085,828-12,089,631 reverse strand. ***(FOXJ2 ortholog)***
--
PHC1.2 scaffold_149: 55,191-61,336 reverse strand.
ENSGACG00000001994 scaffold_149: 66,276-72,088 reverse strand.
(SLC2A3/SLCA14 ortholog)

Xiphophorus maculatus (Platyfish) Scaffold JH556690 and Scaffold JH556782
PEX5 Scaffold JH556690.1: 1,221,752-1,239,972 reverse strand.
//
PHC1.2 Scaffold JH556690.1: 1,816,343-1,826,235 reverse strand.
M6PR Scaffold JH556690.1: 1,827,153-1,832,591 reverse strand.
//
ENSXMAG00000011810 Scaffold JH556690.1: 1,991,922-2,006,008 reverse strand.
(SLC2A3/SLC2A14 ortholog)
//
AICDA Scaffold JH556690.1: 2,090,897-2,093,422 forward strand.
NECAP1.2 Scaffold JH556690.1: 2,093,837-2,103,915 forward strand.

MFAP5 Scaffold JH556690.1: 2,104,822-2,108,627 reverse strand.
ENSXMAG00000011908 Scaffold JH556690.1: 2,120,292-2,126,152 reverse strand. ***(SBK2 ortholog)***
EPN1 Scaffold JH556690.1: 2,129,161-2,147,388 forward strand.
ENSXMAG00000011915 Scaffold JH556690.1: 2,148,990-2,155,492 reverse strand. ***(FOXJ2 ortholog)***
--
ENSXMAG00000000924 Scaffold JH556782.1: 129,400-144,534 forward strand. ***(SLC2A3/SLC2A14 ortholog)***
PHC1.1 Scaffold JH556782.1: 152,283-165,175 forward strand.

Tetraodon nigroviridis (Tetraodon) Chromosome 8 and Chromosome Un_random
M6PR Chromosome 8: 8,262,733-8,263,059 reverse strand.
//
ENSTNIG00000013823 Chromosome 8: 8,331,555-8,334,369 reverse strand. ***(SLC2A3/SLCA14 ortholog)***
//
AICDA Chromosome 8: 8,379,645-8,380,552 forward strand.
NECAP1.1 Chromosome 8: 8,381,268-8,383,762 forward strand.
MFAP5 Chromosome 8: 8,384,808-8,387,112 reverse strand.
ENSTNIG00000008115 Chromosome 8: 8,390,815-8,392,311 reverse strand. ***(SBK2 ortholog)***
EPN1 Chromosome 8: 8,399,230-8,404,144 forward strand.
ENSTNIG00000008113 Chromosome 8: 8,405,675-8,408,111 reverse strand. ***(FOXJ2 ortholog)***
//
PHC1.1 Chromosome 8: 8,981,200-8,984,907 forward strand.
PEX5.1 Chromosome 8: 9,259,224-9,263,479 forward strand.
PEX5.2 Chromosome 8: 9,279,349-9,286,488 forward strand.
--
PHC1.2 Chromosome Un_random: 12,366,576-12,372,990 reverse strand.
//
ENSTNIG00000018188 Chromosome Un_random: 16,452,515-16,455,366 reverse strand. ***(SLC2A3/SLCA14 ortholog)***
//
NECAP1.2 Chromosome Chromosome Un_random: 16,676,326-16,678,444 forward strand.
//
ENSTNIG00000011738 Chromosome Un_random: 18,266,978-18,268,021 forward strand. ***(C3AR1/C5AR1/C5AR2 ortholog)***

Takifugu rubripes (Fugu) scaffold_202 and scaffold_61
FOXJ2 scaffold_202: 252,920-259,014 forward strand.
EPN1 scaffold_202: 260,151-268,711 reverse strand.
ENSTRUG00000006836 scaffold_202: 275,279-276,894 forward strand.
MFAP5 scaffold_202: 260,151-268,711 forward strand ***(Unpredicted, putative)***
NECAP1.2 scaffold_202: 285,254-288,116 reverse strand.

AID scaffold_202: 289,154-290,607 reverse strand. ***(AICDA ortholog)***
//
ENSTRUG00000007710 scaffold_202: 333,209-338,532 forward strand.
(SLC2A3/SLCA14 ortholog)
//
TRU.3523 scaffold_202: 393,138-395,054 forward strand. ***(M6PR ortholog)***
--
ENSTRUG00000005156 scaffold_61: 47,564-53,838 reverse strand.
(SLC2A3/SLCA14 ortholog)
//
NECAP1.1 scaffold_61: 327,906-330,517 forward strand.

Oryzias latipes (Medaka) Chromosome 16 and Chromosome 11
FOXJ2 Chromosome 16: 16,789,561-16,793,142 forward strand.
EPN1 Chromosome 16: 16,794,770-16,804,673 reverse strand.
MFAP5 Chromosome 16: 16,818,234-16,821,428 forward strand.
NECAP1.2 Chromosome 16: 16,822,783-16,828,547 reverse strand.
AICDA Chromosome 16: 16,829,636-16,830,538 reverse strand.
//
ENSORLG00000011838 Chromosome 16: 16,894,851-16,899,958 forward strand.
(SLC2A3/SLCA14 ortholog)
//
M6PR Chromosome 16: 16,999,509-17,002,569 forward strand.
PHC1.2 Chromosome 16: 17,008,042-17,014,271 forward strand.
--
NECAP1.1 Chromosome 11: 13,994,031-14,004,871 reverse strand.
//
ENSORLG00000006093 Chromosome 11: 14,809,067-14,817,034 forward strand.
(SLC2A3/SLCA14 ortholog)
PHC1.1 Chromosome 11: 14,824,205-14,836,443 forward strand.

Gadus morhua (Cod) GeneScaffold_1960
ENSGMOG00000004406 GeneScaffold_1960: 278,080-281,856 reverse strand.
(FOXJ2 ortholog)
//
NECAP1.1 GeneScaffold_1960: 230,327-235,639 forward strand.
AICDA GeneScaffold_1960: 226,520-229,999 forward strand.
//
ENSGMOG00000003970 GeneScaffold_1960: 152,430-159,954 reverse strand.
(SLC2A3/SLCA14 ortholog)
