# Supplementary material for: On the Origin and Evolutionary History of NANOG
Source: PLoS One. 2014 Jan 17;9(1):e85104. doi: 10.1371/journal.pone.0085104 (PMC3894937; doi:10.1371/journal.pone.0085104)
Supplement: Table S1 — Table listing the source of the NANOG sequences used in this study. Coordinates of the genes on chromosomes/genomic scaffolds are given when available. Genes that were either newly predicted or for which novel or revised intron/exons boundaries were identified are listed in red. (DOCX) [file pone.0085104.s005.docx]

| **Gene** | **Species** | **Accession numbers** | **Genomic Location** |
| --- | --- | --- | --- |
| **Nanog1** | *Lepisosteus oculatus* | new prediction based on GenBank:JH591418.1 | unknown |
| **Nanog2** | *Lepisosteus oculatus* | new prediction based on GenBank:JH591418.1 | unknown |
| **Nanog1** | *Salmo salar* | new prediction based on AGKD01009946.1:1..534000 and AGKD01119367.1:1..141000 | unknown |
| **Nanog2** | *Salmo salar* | new prediction based on AGKD01058068.1:10000..17000 | unknown |
| **Nanog** | *Oryzias latipes* | Genbank:ACJ51123.1 | Chromosome 20: 10,394,648-10,399,531 |
| **Nanog** | *Gasterosteus aculeatus* | new prediction based on Ensembl:ENSGACG00000002275 | groupXXI: 3,775,351-3,776,625 |
| **Nanog** | *Gadus morhua* | new prediction based on Ensembl:GeneScaffold_1975 and Ensembl:ENSGMOG00000001786 | GeneScaffold_1975: 25,333-26,467 |
| **Nanog** | *Xiphophorus maculatus* | new prediction based on Ensembl:ENSXMAG00000006084 | Scaffold JH556760.1: 336,167-338,568 |
| **Nanog** | *Dicentrachus labrax* | GenBank:CBN81816.1 | unknown |
| **Nanog** | *Carassius auratus* | new prediction based on Genbank:JF773571.1 and JF773570.1 | unknown |
| **Nanog** | *Danio rerio* | Ensembl:ENSDARG00000075113 | Chromosome 24: 12,738,148-12,743,225 |
| **Nanog** | *Takifugu rubripes* | new prediction based on Ensembl:ENSTRUG00000012825 | Scaffold_95: 962,854-965,761 |
| **Nanog** | *Tetraodon nigroviridis* | new prediction based on Ensembl:ENSTNIG00000005192 | Chromosome 6: 574,356-576,008 |
| **Nanog1** | *Latimeria chalumnae* | new prediction based on Ensembl:Scaffold JH127875.1:345,415-352,530 | Scaffold JH127875.1:345,415-352,530 |
| **Nanog2** | *Latimeria chalumnae* | new prediction based on Ensembl:Scaffold JH127875.1:316,921-323,744 | Scaffold JH127875.1:316,921-323,744 |
| **Nanog** | *Cynops pyrrhogaster* | from maki et al., 2009 | unknown |
| **Nanog** | *Notophthalmus viridescens* | GenBank:ACM89425.1 | unknown |
| **Nanog** | *Ambystoma mexicanum* | GenBank:ADD69772.1 | Linkage Group 3 (LG3) |
| **Nanog1** | *Anolis carolinensis* | Ensembl:ENSACAG00000015317 | Chromosome 2: 80,930,150-80,939,167 |
| **Nanog** | *Thamnophis elegans* | new prediction based on contig71673 length=1925 numreads=87 from http://eco.bcb.iastate.edu/ | unknown |
| **Nanog** | *Python molurus* | new prediction based on GenBank:AEQU010081539.1 and GenBank:AEQU010652512.1 and GenBank:AEQU010652512.1 | unknown |
| **NanogPS** | *Python molurus* | new prediction based on GenBank: AEQU010993990.1 | unknown |
| **Nanog1** | *Chrysemys picta bellii* | Ensembl:ENSPSIG00000012334_1 | Scaffold JH584846.1: 2,538,675-2,545,711 |
| **Nanog2** | *Chrysemys picta bellii* | Ensembl:ENSPSIG00000012067_1 | Scaffold JH584846.1: 2,520,300-2,533,529 |
| **Nanog1** | *Pelodiscus sinensis* | Ensembl:ENSPSIG00000012334 | Scaffold JH207983.1: 235,450-240,970 |
| **Nanog2** | *Pelodiscus sinensis* | Ensembl:ENSPSIG00000012067 | Scaffold JH207983.1: 219,310-230,273 |
| **Nanog1** | *Melopsittacus undulatus* | Ensembl:TGUHOMG00000013513_1 | Scaffold JH556605.1: 7,332,551-7,335,558 |
| **Nanog2** | *Melopsittacus undulatus* | New prédiction based on Ensembl:TGUHOMG00000013515_1 | Scaffold JH556605.1: 7,323,008-7,325,333 |
| **Nanog1** | *Taenopygia guttata* | Ensembl:ENSTGUG00000013123 | Chromosome 1: 88,232,783-88,235,011 |
| **Nanog2** | *Taenopygia guttata* | New prédiction based on Ensembl:ENSTGUG00000013121 | Chromosome 1: 88,224,132-88,226,482 |
| **Nanog1** | *Meleagris gallopavo* | Ensembl:ENSMGAG00000013941 | Chromosome 1: 79,355,894-79,358,232 |
| **Nanog2** | *Meleagris gallopavo* | New prédiction based on Ensembl:ENSMGAG00000016732 | Chromosome 1: 79,347,037-79,348,940 |
| **Nanog1** | *Anas platyrhynchos* | Ensembl:ENSAPLG00000013881 | Scaffold226: 134,950-136,898 |
| **Nanog2** | *Anas platyrhynchos* | EnsemblENSAPLG00000013879 | Scaffold226: 123,906-128,797 |
| **Nanog1** | *Gallus gallus* | GenBank:ABK27429.1 | Chromosome 1: 75,366,745-75,371,263 |
| **Nanog2** | *Gallus gallus* | new prediction based on Ensembl:ENSGALG00000028398.1 | Chromosome 1: 75,344,339-75,363,976 |
| **Nanog1** | *Geospiza fortis* | new prediction based on GenBank:AKZB01048679.1 | unknown |
| **Nanog2** | *Geospiza fortis* | new prediction based on GenBank:AKZB01048679.1 | unknown |
| **Nanog1** | *Ficedula albicollis* | Ensembl:ENSTGUP00000013513_1 | Scaffold JH603217.1: 145,714-148,553 |
| **Nanog2** | *Ficedula albicollis* | New prédiction based onEnsembl:ENSTGUP00000013515_1 | Scaffold JH603217.1: 137,027-139,251 |
| **Nanog1** | *Alligator mississippiensis* | new prediction based on GenBank:AKHW01051965.1 | unknown |
| **Nanog2** | *Alligator mississippiensis* | new prediction based on GenBank:AKHW01051965.1 | unknown |
| **Nanog1** | *Ornithorhynchus anatinus* | New prédiction based on GenBank:LOC100085977 | SuperContig:Contig7532:37,754-41,466 |
| **Nanog2** | *Ornithorhynchus anatinus* | new prediction based on Ensembl:Contig6405:1-20,000 | SuperContig:Contig6405:9,000-20,000 |
| **Nanog1** | *Sarcophilus harrisi* | GenBank:XM_003772450.1 | Scaffold GL861820.1: 100,942-104,884 |
| **Nanog2** | *Sarcophilus harrisi* | new prediction based on Ensembl:GL840998.1:1889-2940 | Scaffold GL840998.1:1889-2940 |
| **Nanog1** | *Monodelphis domestica* | new prediction based on Ensembl:ENSMODG00000017979 | Chromosome 8: 104,628,273-104,629,994 |
| **Nanog1** | *Macropus eugenii* | new prediction based on Ensembl:ENSMEUG00000009949 | Scaffold11284: 12,337-13,800 |
| **Nanog** | *Mus musculus* | Ensembl:ENSMUSG00000012396 | Chromosome 6: 122,657,507-122,664,651 |
| **Nanog1** | *Cavia porcellus* | Ensembl:ENSCPOG00000008888 | scaffold_28: 2,167,077-2,172,849 |
| **Nanog2** | *Cavia porcellus* | new prediction based on Ensembl:ENSCPOG00000002621 | scaffold_28: 2,498,785-2,504,514 |
| **Nanog** | *Oryctolagus cuniculus* | Ensembl:ENSOCUG00000013783 | Chromosome 8: 33,789,671-33,797,889 |
| **Nanog** | *Tupaia belangeri* | New prédiction based on Ensembl:ENSTBEG00000000557 | GeneScaffold_4682: 49,136-51,623 |
| **Nanog1** | *Homo sapiens* | Ensembl:ENSG00000111704 | Chromosome 12: 7,940,390-7,948,655 |
| **NanogGP1** | *Homo sapiens* | Ensembl:ENSG00000176654 | Chromosome 12: 8,025,534-8,052,674 |
| **Nanog1** | *Pan troglodytes* | Ensembl:ENSPTRG00000029807 | Chromosome 12: 8,021,059-8,027,419 |
| **NanogGP1** | *Pan troglodytes* | new prediction based on Ensembl:Chromosome 12: 8,121,100-8,136,565 | Chromosome 12: 8,121,100-8,136,565 |
| **Nanog** | *Canis lupus familiaris* | Ensembl:ENSCAFG00000013914 | Chromosome 27: 37,260,380-37,266,023 |
| **Nanog** | *Ailuropoda melanoleuca* | Ensembl:ENSAMEG00000017064 | Scaffold GL194054.1: 275,383-279,705 |
| **NanogPS** | *Ailuropoda melanoleuca* | Ensembl:ENSAMEG00000006432 | Scaffold GL192340.1: 5,244,108-5,244,955 |
| **Nanog** | *Felis catus* | Ensembl:ENSFCAG00000001621 | Chromosome B4: 45,132,294-45,137,356 |
| **NanogPS1** | *Felis catus* | Ensembl:ENSFCAG00000029654 | Chromosome C1: 75,183,500-75,184,521 |
| **NanogPS2** | *Felis catus* | Ensembl:ENSFCAG00000026166 | Chromosome C1: 76,305,440-76,306,084 |
| **Nanog** | *Sus scrofa* | Ensembl:ENSSSCG00000004977 | Chromosome 1: 187,711,367-187,712,525 |
| **Nanog** | *Bos Taurus* | Ensembl:ENSBTAG00000020916 | Chromosome 5: 101,871,073-101,876,226 |
| **Nanog** | *Myotis lucifugus* | Ensembl:ENSMLUG00000028740 | Scaffold GL429777: 9,038,390-9,043,290 |
| **NanogPS** | *Myotis lucifugus* | Ensembl:ENSMLUG00000029325 | Scaffold GL431244: 34,777-40,205 |
| **Nanog** | *Pterobat vampirus* | Ensembl:ENSPVAG00000006989 | scaffold_8071: 64,939-68,863 |
| **Nanog** | *Equus caballus* | Ensembl:ENSECAG00000012614 | Chromosome 6: 35,483,581-35,487,984 |
| **Nanog** | *Loxodonta africana* | Ensembl:ENSLAFG00000012934 | SuperContig scaffold_15: 53,444,918-53,449,716 |
| **Nanog** | *Procavia capensis* | new prediction based on Ensembl:ENSPCAG00000015460 | scaffold_21276: 8,690-10,666 |
| **Nanog** | Choloepus hoffmanni | Ensembl:ENSCHOG00000000972 | scaffold_14792: 5,055-10,053 |
