# Supplementary material for: On the Origin and Evolutionary History of NANOG
Source: PLoS One. 2014 Jan 17;9(1):e85104. doi: 10.1371/journal.pone.0085104 (PMC3894937; doi:10.1371/journal.pone.0085104)
Supplement: Figure S1 — Alignment used for the phylogenetic analysis. The regions encoded by the 2nd and 3rd exons of the NANOG genes, as well the homeodomain are indicated below the alignment. (PDF) [file pone.0085104.s001.pdf]

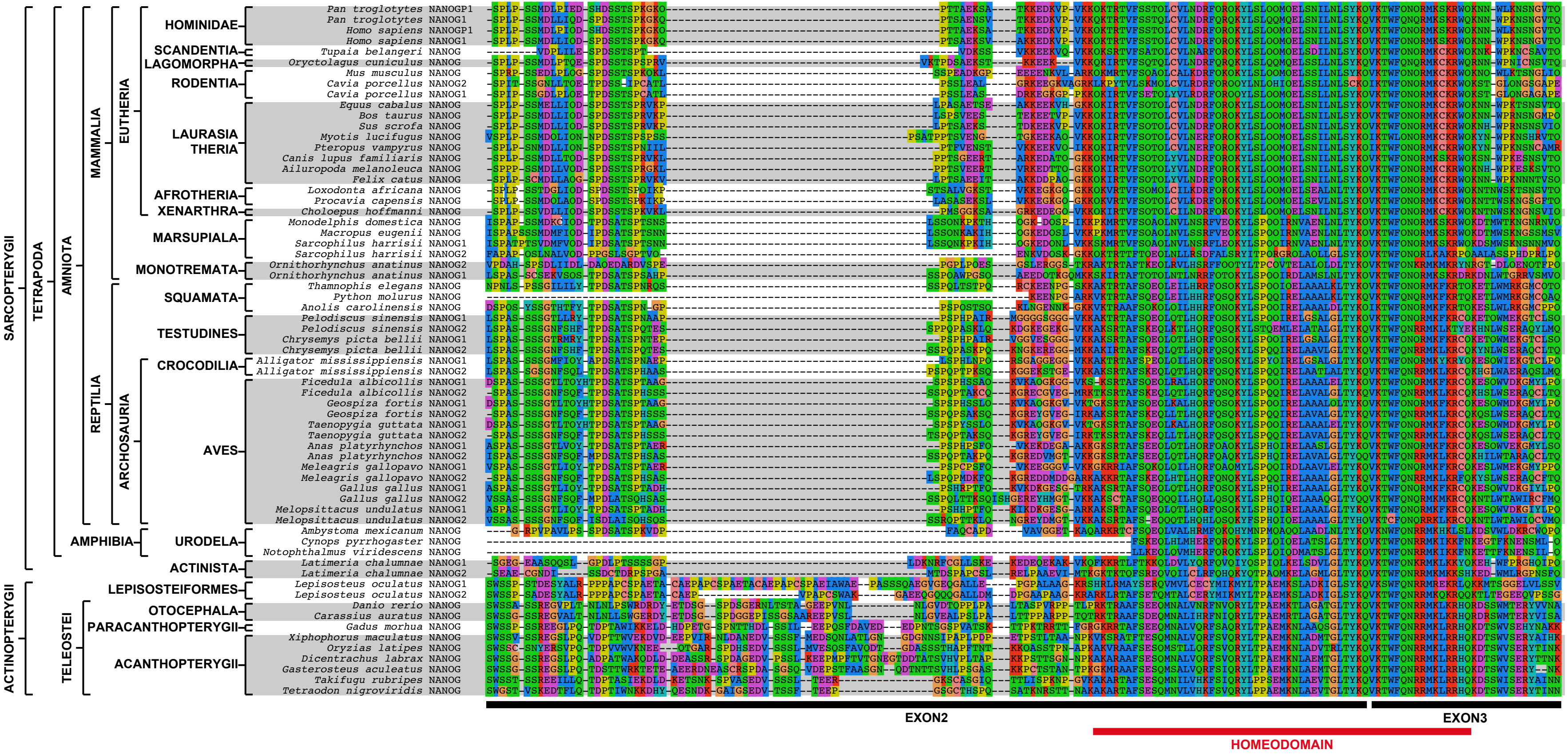
SARCOPTERYGII
TETRAPODA
AMNIOTA
MAMMALIA
EUTHERIA
HOMINIDAE
Pan troglotytes NANOGP1
Pan troglotytes NANOG1
Homo sapiens NANOGP1
Homo sapiens NANOG1
SCANDENTIA
Tupaia belangeri NANOG
LAGOMORPHA
Oryctolagus cuniculus NANOG
RODENTIA
Mus musculus NANOG
Cavia porcellus NANOG2
Cavia porcellus NANOG1
LAURASIA THERIA
Equus cabalus NANOG
Bos taurus NANOG
Sus scrofa NANOG
Myotis lucifugus NANOG
Pteropus vampyrus NANOG
Canis lupus familiaris NANOG
Ailuropoda melanoleuca NANOG
Felix catus NANOG
AFROTHERIA
Loxodonta africana NANOG
Procavia capensis NANOG
XENARTHRA
Choloepus hoffmanni NANOG
MARSUPIALA
Monodelphis domestica NANOG
Macropus eugenii NANOG
Sarcophilus harrisii NANOG1
Sarcophilus harrisii NANOG2
MONOTREMATA
Ornithorhynchus anatinus NANOG2
Ornithorhynchus anatinus NANOG1
REPTILIA
SQUAMATA
Thamnophis elegans NANOG
Python molurus NANOG
Anolis carolinensis NANOG
TESTUDINES
Pelodiscus sinensis NANOG1
Pelodiscus sinensis NANOG2
Chrysemys picta bellii NANOG1
Chrysemys picta bellii NANOG2
ARCHOSAURIA
CROCODILIA
Alligator mississippiensis NANOG1
Alligator mississippiensis NANOG2
AVES
Ficedula albicollis NANOG1
Ficedula albicollis NANOG2
Geospiza fortis NANOG1
Geospiza fortis NANOG2
Taenopygia guttata NANOG1
Taenopygia guttata NANOG2
Anas platyrhynchos NANOG1
Anas platyrhynchos NANOG2
Meleagris gallopavo NANOG1
Meleagris gallopavo NANOG2
Gallus gallus NANOG1
Gallus gallus NANOG2
Melopsittacus undulatus NANOG1
Melopsittacus undulatus NANOG2
AMPHIBIA
URODELA
Ambystoma mexicanum NANOG
Cynops pyrrhogaster NANOG
Notophthalmus viridescens NANOG
ACTINISTA
Latimeria chalumnae NANOG1
Latimeria chalumnae NANOG2
ACTINOPTERYGII
LEPISOSTEIFORMES
Lepisosteus oculatus NANOG1
Lepisosteus oculatus NANOG2
TELEOSTEI
OTOCEPHALA
Danio rerio NANOG
Carassius auratus NANOG
PARACANTHOPTERYGII
Gadus morhua NANOG
ACANTHOPTERYGII
Xiphophorus maculatus NANOG
Oryzias latipes NANOG
Dicentrachus labrax NANOG
Gasterosteus aculeatus NANOG
Takifugu rubripes NANOG
Tetraodon nigroviridis NANOG
EXON2
EXON3
HOMEODOMAIN
